# Lack of the COMPASS Component Ccl1 Reduces H3K4 Trimethylation Levels and Affects Transcription of Secondary Metabolite Genes in Two Plant–Pathogenic *Fusarium* Species

**DOI:** 10.3389/fmicb.2016.02144

**Published:** 2017-01-09

**Authors:** Lena Studt, Slavica Janevska, Birgit Arndt, Stefan Boedi, Michael Sulyok, Hans-Ulrich Humpf, Bettina Tudzynski, Joseph Strauss

**Affiliations:** ^1^Division of Microbial Genetics and Pathogen Interactions, Department of Applied Genetics and Cell Biology, BOKU-University of Natural Resources and Life SciencesVienna, Tulln an der Donau, Austria; ^2^Institute for Plant Biology and Biotechnology, Westfälische Wilhelms UniversityMünster, Germany; ^3^Institute of Food Chemistry, Westfälische Wilhelms UniversityMünster, Germany; ^4^Center for Analytical Chemistry, Department IFA-Tulln, BOKU-University of Natural Resources and Life SciencesVienna, Tulln an der Donau, Austria

**Keywords:** *Fusarium*, H3K4 methylation, COMPASS, secondary metabolism, virulence, histone modification

## Abstract

In the two fungal pathogens *Fusarium fujikuroi* and *Fusarium graminearum*, secondary metabolites (SMs) are fitness and virulence factors and there is compelling evidence that the coordination of SM gene expression is under epigenetic control. Here, we characterized Ccl1, a subunit of the COMPASS complex responsible for methylating lysine 4 of histone H3 (H3K4me). We show that Ccl1 is not essential for viability but a regulator of genome-wide trimethylation of H3K4 (H3K4me3). Although, recent work in *Fusarium* and *Aspergillus* spp. detected only sporadic H3K4 methylation at the majority of the SM gene clusters, we show here that SM profiles in *CCL1* deletion mutants are strongly deviating from the wild type. Cross-complementation experiments indicate high functional conservation of Ccl1 as phenotypes of the respective △*ccl1* were rescued in both fungi. Strikingly, biosynthesis of the species-specific virulence factors gibberellic acid and deoxynivalenol produced by *F. fujikuroi* and *F. graminearum*, respectively, was reduced in axenic cultures but virulence was not attenuated in these mutants, a phenotype which goes in line with restored virulence factor production levels *in planta.* This suggests that yet unknown plant-derived signals are able to compensate for Ccl1 function during pathogenesis.

## Introduction

Filamentous fungi produce a huge arsenal of secondary metabolites (SMs), small molecular-weight compounds that are not needed for the survival of the fungus but are thought to contribute to the fitness of its producer in some way (Fox and Howlett, 2008; Reverberi et al., 2010; Rohlfs and Churchill, 2011). Among those SMs are potent toxins that frequently contaminate food and feed (Streit et al., 2013), but also pharmaceuticals and plant hormones that are applied in medicine and agriculture, respectively (Dreyfuss et al., 1976; Anke et al., 1977; Rademacher, 1997; Manzoni and Rollini, 2002). SM production is energy consuming, and thus, only initiated when advantageous for the producer. This implies a tight regulatory network functioning in induction or repression of the respective SM genes. Genes involved in SM biosynthesis are generally clustered (Keller and Hohn, 1997), and thus, prone to chromatin level regulation. The chromatin landscape is either loosely packed, open for transcription (euchromatin) or densely packed and silenced (heterochromatin). Both chromatin states are associated with the activity of certain histone-modifying enzymes that are often part of large enzyme complexes and involved in the addition or removal of residues at defined amino acids of the histone protein tails (e.g., Bannister and Kouzarides, 2011; Gacek and Strauss, 2012).

One such complex is COMPASS (complex of proteins associated with Set1) that is highly conserved from yeast to humans and plants (Miller et al., 2001; Krogan et al., 2002; Hughes et al., 2004; Lee et al., 2007; Wu et al., 2008; Jiang et al., 2011; Shilatifard, 2012). The catalytic subunit, the methyltransferase Set1 (Kmt2), was first identified and co-purified in *Saccharomyces cerevisiae* together with a complex consisting of eight subunits, i.e., Set1, Bre2, Swd1, Spp1, Swd2, Swd3, Sdc1, and Shg1 (Nislow et al., 1997; Miller et al., 2001; Roguev et al., 2001). Set1 catalyzes mono-, di-, and trimethylation of the fourth lysine at histone 3 (H3K4me1/2/3) (Roguev et al., 2001; Krogan et al., 2002; Liu et al., 2015). However, Set1 alone is not functional as each of the subunits has a specific function in complex assembly and integrity, pattern of global H3K4 methylation and distribution of H3K4me along active genes (Dehé et al., 2006; Shilatifard, 2012). One member of COMPASS is Bre2 (Ash2 in *Schizosaccharomyces pombe* and *Magnaporthe oryzae*, CclA in *Aspergillus* spp.) that is specifically involved in H3K4me3 with limited to no effect on di- and no effect on monomethylation (Schlichter and Cairns, 2005; Schneider et al., 2005; Dehé et al., 2006; Bok et al., 2009; Palmer et al., 2013; Mikheyeva et al., 2014; Pham et al., 2015). Notably, while H3K4 methylation is generally associated with transcriptional gene activation, COMPASS-mediated silencing of genes located near chromosome telomeres has also been described (Briggs et al., 2001; Krogan et al., 2002; Bok et al., 2009; Mikheyeva et al., 2014). Deletion of the *bre2* homolog *cclA* in *Aspergillus* spp. resulted in increased expression of several SM cluster genes (Bok et al., 2009; Palmer et al., 2013; Shinohara et al., 2016).

In this study, we analyzed the influence of the *bre2* homolog *CCL1* in two *Fusarium* spp., that is the rice pathogen *Fusarium fujikuroi* (teleomorph *Gibberella fujikuroi*) responsible for *bakanae* disease on rice (Wiemann et al., 2013), and the cereal crop pathogen *Fusarium graminearum* (teleomorph *Gibberella zeae*) causing severe epidemics of head blight (Kazan et al., 2012). These two fungi are able to produce numerous SMs. Notably most of the SMs are species-specific and produced by only one of the two fusaria. Prominent examples are the natural plant hormone gibberellins (GAs) and the mycotoxin deoxynivalenol (DON) produced by *F. fujikuroi* and *F. graminearum*, respectively, which are both directly associated with the disease symptoms caused by these two fungi (Proctor et al., 1995; Jansen et al., 2005; Wiemann et al., 2013). In addition, *F. fujikuroi* and *F. graminearum* produce similar but distinct polyketide synthase (PKS)-derived pigments that are thought to function as protectants against environmental stress, i.e., the mycelial pigments bikaverin (BIK) and aurofusarin (AUR), respectively (Malz et al., 2005; Wiemann et al., 2009). Several additional SMs have been characterized in both fungi over the last years. Those include distinct SMs produced by only one of the two fusaria such as fusaric acid (FSA), fumonisins, fujikurins, or apicidin F produced by *F. fujikuroi* (von Bargen et al., 2013, 2015; Niehaus et al., 2014a; Rösler et al., 2016), and zearalenone (ZON), fusarielins, culmorin, or butenolide produced by *F. graminearum* (Hansen et al., 2012; Sørensen et al., 2012). There are also common SMs, such as the fusarins (FUS), a family of structurally related polyketide mycotoxins (Gelderblom et al., 1984; Díaz-Sánchez et al., 2012; Niehaus et al., 2013), and some non-ribosomal peptide synthetase (NRPS)-derived siderophores (Hansen et al., 2015). In this study, we show that the COMPASS component-encoding gene *CCL1* is a conserved yet not essential gene in the two *Fusarium* spp. analyzed, but that deletion had a drastic effect on the SM profiles.

## Materials and Methods

### Fungal Strains, Media, and Growth Conditions

The wild type strain *F. fujikuroi* IMI58289 (Commonwealth Mycological Institute, Kew, UK) as well as the *F. graminearum* wild type strain PH-1 (FGSC 9075, NRRL 31084) were used as parental strains for knock-out experiments. For SM analysis and ChIP experiments, *F. fujikuroi* strains were pre-incubated for 72 h in 300 mL Erlenmeyer flasks with 100 mL Darken medium (DVK) (Darken et al., 1959) on a rotary shaker in constant darkness at 180 rpm and 28°C. A 500 μL aliquot of this culture was used for inoculation of synthetic ICI (Imperial Chemical Industries, Ltd, UK) medium (Geissman et al., 1966), with varying nitrogen sources, either 6 mM (nitrogen starvation) or 60 mM (nitrogen surplus) glutamine. Incubation was carried on for additional three to 7 days in case of chromatin immunoprecipitation (ChIP) analyses and chemical analyses, respectively. *F. graminearum* strains were grown for 2 weeks on solid potato dextrose agar (PDA; Sigma-Aldrich, Germany) for SM quantification. For gene expression analysis, the wild type strain was grown for up to 2 weeks on solid PDA and for ChIP analysis, strains were grown for 4 days on PDA. For protoplasting of *F. fujikuroi*, 500 μL of the pre-incubated culture was taken and transferred into 100 mL ICI medium with 10 g/L fructose instead of glucose and 1 g/L (NH_4_)_2_SO_4_ as nitrogen source and incubated on a rotary shaker at 28°C and 180 rpm no longer than 16 h. For protoplasting of *F. graminearum*, YPG medium (Brown et al., 2002) was inoculated with ∼10^6^ conidia and incubated overnight on a rotary shaker at 24°C and 180 rpm. For conidiation, *F. graminearum* strains were grown for 7 days in liquid carboxymethylcellulose media (CMC) on a rotary shaker at 24°C and 180 rpm (Cappellini and Peterson, 1965). In case of *F. fujikuroi*, solidified vegetable juice (V8) medium was inoculated with 5 mm agar plaques and incubated for 7 days at room temperature. For DNA isolation and western blot analysis, the fungal strains were grown on solidified complete medium (CM) (Pontecorvo et al., 1953) covered with cellophane sheets for 3 days at 28°C in case of *F. fujikuroi* or 20°C in case of *F. graminearum*. Hyphal growth of both fungi was assessed on solid CM as rich media, while *Fusarium* minimal medium (FMM) (Reyes-Dominguez et al., 2012) was chosen as a minimal medium. Plates were incubated for 5 days at 28 and 20°C in case of *F. fujikuroi* and *F. graminearum*, respectively.

### Plasmid Constructions

Plasmids for *F. fujikuroi* and *F. graminearum* knock-out, complementation and cross-complementation strains were generated using yeast recombinational cloning (Colot et al., 2006). All primers used for polymerase chain reaction (PCR) were obtained from Eurofins GmbH (Ebersberg, Germany). For knock-out constructs, the upstream (5′) and downstream (3′) sequences of *FfCCL1* and *FgCCL1* were amplified from *F. fujikuroi* IMI58289 and *F. graminearum* PH-1 genomic DNA, respectively, with the following primer pairs: FfCCL1_5F and FfCCL1_5R for upstream and FfCCL1_3F and FfCCL1_3R for downstream regions in case of *F. fujikuroi* and FgCCL1_5F and FgCCL1_5R for upstream and FgCCL1_3F and FgCCL1_3R for downstream regions in case of *F. graminearum*. In both cases, hygromycin B was used as resistance marker. The hygromycin B resistance cassette, consisting of the hygromycin B phosphotransferase gene *hph* (Gritz and Davies, 1983), driven by the *trpC* promoter, was amplified using the primer pair hph-F and hph-R from the template pCSN44 (Staben et al., 1989). The *S. cerevisiae* strain FY834 was transformed with the obtained fragments (Winston et al., 1995), as well as with the *Eco*RI/*Xho*I restricted plasmid pRS426 (Christianson et al., 1992) yielding plasmids pΔ*ffccl1* and pΔ*fgccl1*. For complementation of Δ*ffccl1*, the respective gene including about 1 kb of the upstream region was amplified using a proof-reading polymerase and the primer pair FfCCL1_5F and FfCCL1C_IL_R. The gene was fused to the glucanase terminator sequence, *BcTgluc*, obtained from *Botrytis cinerea* B05.10 using a proof-reading polymerase and the primer pair BcGlu-term-F2 and Tgluc-nat1-R, followed by the nourseothricin resistance cassette, *natR*, itself driven by the *trpC* promoter. The nourseothricin resistance cassette was amplified with the primer pair Hph-R-nat1 and Hph-F using a proof-reading polymerase and the template pZPnat1 (GenBank accession no. AY631958.1). The downstream region of *FfCCL1* was amplified using the same primer pair as in the deletion approach, i.e., FfCCL1_3F and FfCCL1_3R. *S. cerevisiae* FY834 was transformed with the obtained fragments as well as with the *Eco*RI/*Xho*I restricted pRS426 yielding plasmid p*FfCCL1*^Cil^ (Supplementary Figure S1A). Similarly, complementation of Δ*fgccl1* was accomplished by amplification of *FgCCL1* and about 1 kb upstream region using the primer pair FgCCL1_IL_F and FgCCL1_Tgluc_IL_R as well as a proof-reading polymerase. The gene was fused to the *BcTgluc* terminator sequence followed by the geniticin resistance cassette, *genR*, which was amplified from pKS-Gen (Bluhm et al., 2008) using a proof-reading polymerase and the primer pair Geni-F and Geni-Tgluc-R. The downstream region of *FgCCL1* was amplified from *F. graminearum* PH-1 genomic DNA using the primer pair FgCCL1_3F and FgCCL1_3R. *S. cerevisiae* FY834 was transformed with the obtained fragments as well as with the *Eco*RI/*Xho*I restricted pRS426 yielding plasmid p*FgCCL1*^Cil^ (Supplementary Figure S1A).

For cross-complementation of *FgCCL1* in Δ*ffccl1*, *FgCCL1* was amplified from *F. graminearum* PH-1 genomic DNA using a proof-reading polymerase with the primer pair FgCCL1-FfCCL1_F and FgCCL1-IL_TglucR. The gene was driven by the *FfCCL1* native promoter that was amplified from *F. fujikuroi* IMI58289 genomic DNA using the primer pair FfCCL1_5F and FfCCL1_5R2, and fused to *BcTgluc* followed by the nourseothricin resistance cassette, *nat1R*, as described earlier. Downstream region was amplified from *F. fujikuroi* IMI58289 genomic DNA using the primer pair FfCCL1_3F and FfCCL1_3R. *S. cerevisiae* FY834 was transformed with the obtained fragments as well as with the *Eco*RI/*Xho*I restricted pRS426 yielding plasmid pΔ*ffccl1/FgCCL1* (Supplementary Figure S1B). For cross-complementation of *FfCCL1* in Δ*fgccl1*, the *F. fujikuroi FfCCL1* gene was amplified from *F. fujikuroi* IMI58289 genomic DNA with the primer pair FfCCL1-IL_F and FfCCL1-Tgluc-IL_R using a proof-reading polymerase. The gene was fused to *BcTgluc* followed by the geneticin resistance cassette, *genR*. Upstream and downstream regions were amplified using the primer pairs FgCCL1_5F and FgCCL1-FfCCL1_5R and FgCCL1_3F and FgCCL1_3R, respectively. *S. cerevisiae* FY834 was transformed with the obtained fragments as well as with the *Eco*RI/*Xho*I restricted pRS426 yielding plasmid pΔ*fgccl1/FfCCL1* (Supplementary Figure S1B). Correct assembly of the resulting vectors, p*FfCCL1*^Cil^, p*FgCCL1*^Cil^, pΔ*ffccl1/FgCCL1* and pΔ*fgccl1/FfCCL1* was verified by sequencing.

### Fungal Transformations

For generation of *F. fujikuroi* mutants, protoplasts were prepared from the wild type strain IMI58289. Transformation was carried out as previously described (Tudzynski et al., 1996). About 10^7^ protoplasts were transformed with about 10 μg of the amplified replacement cassettes for generating the knock-outs. The knock-out fragments were amplified prior to transformation using pΔ*ffccl1*, the primer pair FfCCL1_5F and FfCCL1_5R and TAKARA polymerase. In case of *F. graminearum*, we used the split-marker approach (Goswami, 2012). For this, two knock-out fragments containing about two thirds of *hph* each were amplified from pΔ*fgccl1* using the primer pairs FgCCL1_5F and split-mark hph_F as well as split-mark hph_R and FgCCL1_3R. Transformation of *F. graminearum* was according to *F. fujikuroi* with minor adjustments. For complementation and cross-complementation of Δ*ffccl1*, the deletion mutant Δ*ffccl1*_T9 was transformed with 10 μg of the respective plasmid p*FfCCL1*^Cil^ and pΔ*ffccl1/FgCCL1*. Plasmids were linearized with either *Pvu*II or *Ssp*I prior to transformation (Supplementary Figure S1). Complementation and cross-complementation of Δ*fgccl1* was performed according to the split-marker approach (Goswami, 2012). Therefore, knock-out fragments were amplified with the following primer pairs: FgCCL1_5F and genR_splitF as well as genR_splitR and FgCCL1_3R. All transformed protoplasts were regenerated as described (Tudzynski et al., 1999). The medium contained 100 ppm of the appropriate resistance marker. Single conidial cultures were established from either hygromycin B-, nourseothricin-, or geneticin-resistant transformants and used for subsequent DNA isolation. In case of Δ*ffccl1* and Δ*fgccl1* mutants, homologous integration of the resistance cassette was verified using the primer pairs dia_FfCCL1_5′ and TrpC-T for the upstream part as well as dia_FfCCL1_3′ and TrpC-P for the downstream part in case of *F. fujikuroi* and dia_FgCCL1_5′ and TrpC-T (upstream) as well as dia_FgCCL1_3′ and TrpC-P (downstream) in case of *F. graminearum*. Absence of the wild type gene was verified using the primer pairs FfCCL1_WT-F and FfCCL1_WT-R as well as FgCCL1_WT_F and FgCCL1_WT_R in case of *F. fujikuroi* and *F. graminearum*, respectively (Supplementary Figure S2). Homologous integration of the *F. fujikuroi* (cross-) complementation constructs was verified using the primer pairs dia_FfCCL1_5′ and FfCCL1_Seq1 (complementation), dia_FfCCL1_5′ and FgCCL1_IL_dia (cross-complementation) for the upstream parts as well as dia_FfCCL1_3′//Nat1_R1 for the downstream parts. Accordingly, homologous integration in *F. graminearum* was verified using the primer pairs dia_ILCom_FgCCL1_5F and dia_ILCom_FgCCL1_5R (complementation) as well as dia_ILCom_FgCCL1_5F and dia_IL-CCom_FgxFfCCL1_5R (cross-complementation) for the upstream region as well as gen-Seq3 and dia_IL-Com_FgCCL1_3R for the downstream region. Absence of *hph* was checked by phenotypic characterization of the complemented mutants, thus, absence of growth in the presence of hygromycin B, as well as by diagnostic PCR using the primer pair dia_FfCCL1_5′ and TrpC-T in case of *F. fujikuroi* as well as dia_IL_Com_FgCCL1_5F and trpC-T in case of *F. graminearum* (Supplementary Figure S3).

### Standard Molecular Techniques

For DNA isolation of both fungi, lyophilized mycelium was ground to a fine powder and resuspended in extraction buffer according to Cenis (1992). Isolated DNA was used for PCR amplification and Southern blot analysis. In case of diagnostic PCR, PCR reactions were set up as follows: 25 ng genomic DNA, 5 pmol of each primer, 200 nM deoxynucleoside triphosphates, and 1 unit BioTherm^TM^ DNA polymerase (GeneCraft GmbH, Lüdinghausen, Germany). Reactions were performed with an initial denaturating step at 94°C for 3 min followed by 35 cycles of 1 min at 94°C, 1 min at 56–60°C, 1 min/kb at 70°C and a final elongation step at 70°C for 10 min. Amplification of knock-out, complementation and cross-complementation constructs was accomplished using a proof-reading polymerase. Therefore, PCR reactions contained 25 ng genomic DNA, 5 pmol of each primer, and 1 unit of Phusion^®^ polymerase (Finnzymes, Thermo Fisher Scientific, Finland). Plasmid DNA from *S. cerevisiae* was extracted using the yeast plasmid isolation kit (SpeedPrep, DualsystemsBioTech) and directly used for PCR reaction. In case of knock-out mutants, *F. fujikuroi* and *F. graminearum* genomic DNA that was subsequently applied for Southern blot analysis. Therefore, genomic DNA was digested with *Sac*I and *Xba*I, respectively, separated on a 1% (w/v) agarose gel and transferred onto nylon membranes (Nytran^®^ SPC, Whatman) by downward blotting (Ausubel et al., 1987). Then, ^32^P-labeled probes were generated using the random oligomer-primer method and hybridized to the membranes overnight at 65°C according to the protocol of (Sambrook et al., 1989) and subsequently washed with 1x SSPE (0.18 M NaCl, 10 mM NaH_2_PO_4_ and 1 mM EDTA, pH 7), 0.1% (w/v) SDS at the same temperature. In case of *F. fujikuroi*, the downstream region amplified with the primer pair FfCCL1_3F and FfCCL1_3R was used for probing (Supplementary Figure S2). For *F. graminearum*, *hph* was used for probing in order to exclude multiple integrations of both transformed knock-out fragments. The *hph* cassette was amplified using the primer pair hph_F and hph_R. RNA was extracted using TRIzol Reagent (Invitrogen^TM^) according to the manufacturer’s instructions. For cDNA synthesis, 1 μg of total RNA was DNase I treated (Fermentas, Schwerte, Germany) and reverse transcribed using the oligo (dT) 12–18 primer and SuperScript II Polymerase [Invitrogen (Life Technologies), Darmstadt, Germany] as described by the manufacturer. For western blot analysis, mycelium from 3 days-old strains was ground to a fine powder with liquid nitrogen and proteins were extracted using a modified TCA (trichloroacetic acid) protocol from Reyes-Dominguez et al. (2010). Briefly, about 200 mg per sample was resuspended in 1 mL TCA (12%, v/v) vortexed vigorously and kept on ice for 10 min prior to centrifugation (10 min, 4°C, 2015 × *g*). The supernatant was discarded and the pellet was washed three times in 1 mL aceton followed by centrifugation (5 min, 4°C, 2015 × *g*). The pellet was dried at 65°C and finally extracted with 500 μL extraction buffer (100 mM Tris pH 8, 50 mM NaCl, 1% SDS, 1 mM EDTA pH 8) with protease inhibitor cocktail (Sigma-Aldrich, St. Louis, MO, USA) in a dilution of 1:200. After centrifugation, the protein concentration of the supernatant was quantified using the BCA protein quantification kit (Pierce, USA). Roughly, 15 μg of proteins were used for SDS-Page and subsequent western blotting. The membrane was probed with H3 C-Term (Active Motif AM39163), H3K4me1 (Active Motif AM39297), H3K4me2 (Active Motif AM39141) as well as three different H3K4me3 (Active Motif AM39159, abcam ab8580, Millipore MP#07-473) primary antibodies and anti-rabbit (Sigma A0545) HRP conjugated secondary antibody. Chemiluminescence was detected with Clarity^TM^ ECL Western Substrate and ChemDoc^TM^ XRS (Bio-Rad).

### Pathogenicity Assays

*F. fujikuroi* pathogenicity assays were performed according to Wiemann et al. (2010). Briefly, germinated rice seedlings (*Oryza sativa* sp. *japonica* cv. Nipponbare) were inoculated with the *F. fujikuroi* wild type strain and a Δ*ffccl1* mutant. 100 ppm GA3 and H_2_O served as a positive and negative control, respectively. After 7 days at 28°C, 80% humidity and a 12 h/12 h light–dark cycle, rice plants were screened for typical *bakanae* disease symptoms. For quantification of GA3 *in planta*, experiments were performed in quintuplicates and rice plants were combined before extraction. Non-infected rice seedlings served as a negative control. For *F. graminearum* pathogenicity assays, the USU-Apogee full-dwarf hard red spring wheat (*Triticum aestivum* cv. USU-Apogee; Reg.no CV-840, PI592742) cultivar was used (Bugbee and Koerner, 1997). Two spikelets per wheat head were inoculated with 1,000 conidia each during anthesis. Mock ears were inoculated using water instead of spore suspension. Five ears per *Fusarium* strain were inoculated and afterward, the wheat heads on the living plants were covered with moistened plastic bags for the first 24 h to provide high humidity. Incubation conditions were set to 50% humidity and 20°C during day-/nighttime and 12 h photoperiod. For quantification of DON *in planta*, harvesting was performed 21 days after infection and plant material was combined prior to extraction.

### Secondary Metabolite Analysis

For *F. fujikuroi*, analysis of all here studied SMs was accomplished using the culture fluids of 7 days-old cultures that were filtered over a 0.45 μm membrane filter (Millex^®^, Millipore) and directly used for analysis without further preparation. *F. graminearum* strains were grown for 2 weeks on PDA agar and subsequently extracted using MeOH/CH_2_Cl_2_/EtOAc (1/2/3, v/v), evaporated and resuspended in acetonitrile/H_2_O (1/1, v/v). In case of *F. graminearum*, samples were run on a QTrap 5500 LC–MS/MS System (Applied Biosystems, Foster City, CA, USA) equipped with a TurboIonSpray electrospray ionization (ESI) source and a 1290 Series high performance liquid chromatography (HPLC) System (Agilent, Waldbronn, Germany). Chromatographic separation was done at 25°C on a Gemini^®^ C18-column, 150 × 4.6 mm i.d., 5 μm particle size, equipped with a C18 4 mm × 3 mm i.d. security guard cartridge (Phenomenex, Torrance, CA, USA). The chromatographic method, chromatographic and mass spectrometric parameters are described elsewhere (Malachová et al., 2014). In case of *F. fujikuroi*, BIK biosynthesis was determined as described elsewhere (Michielse et al., 2014). For FSA, FUS and GA3 measurement, the described HPLC coupled to tandem mass spectrometry (HPLC–MS/MS) method was transferred to a QTrap 6500 mass spectrometer (AB Sciex, Darmstadt, Germany) (Michielse et al., 2014). Data acquisition was performed with Analyst 1.6.2 (AB Sciex). Briefly, for FUS and GA3 analysis, 10 μL of the culture filtrate was mixed with 10 μL methyl parabene (MePa, 10 μg/mL) and the solution was diluted with 980 μL water, resulting in 0.1 μg/mL MePa. For FSA, 10 μL of the culture filtrate was diluted with 990 μL H_2_O and 10 μL of this solution was mixed with MePa as described above. Chromatographic separation was carried out on a 150 mm × 2.1 mm i.d., 5 μm, Eclipse XDB-C18 column (Agilent Technologies, Waldbronn, Germany) with methanol + 1% (v/v) formic acid (FA) as solvent A and water + 1% (v/v) FA as solvent B. The column was tempered to 40°C. After isocratic running for 3 min at 15% A, the gradient rose up to 100% A in 10 min. With the concentration of solvent A, the flow rate also rose from 400 to 450 μL/min. After holding these conditions for 2 min, the column was equilibrated for 3 min. A divert valve was used to discard polar substances from the medium in the first 3 min of the run. For MS/MS analysis with ESI, the following parameters were used: the curtain gas (nitrogen) was set to 30 psi, the nebulizer gas (zero-grade air) to 35 psi and the drying gas (zero-grade air) to 40 psi. The ion spray voltage was set to +5500 V in the positive and to -4500 V in the negative mode. Nitrogen was also used as collision gas in medium mode.

FUS was analyzed with a declustering potential (DP) of +160 V, an entrance potential (EP) of +10 V and a collision energy (CE) of +27 V. The cell exit potential (CXP) was adapted for every transition as described below. Dwell times were set to 20 ms. As parent ion for FUS analysis, the sodium adduct of FUS was used with the following transitions: quantifier FUS (M+Na)^+^: *m*/*z* 454.1→290.1 (CXP +26 V), qualifier 1 FUS: *m*/*z* 454.1→335.1 (CXP +24 V), qualifier 2 FUS: *m*/*z* 454.1→267.2 (CXP +20 V).

FSA was analyzed with a CXP of +10 V, a DP of +40 V and an EP of +10 V. Dwell times were set to 20 ms. Quantifier FSA (M+H)^+^: *m/z* 180.2→134.1 (CE +23 V), qualifier 1 FSA: *m/z* 180.2→91.9 (CE +35 V), qualifier 2 FSA: *m/z* 180.2→65.1 (CE +49 V).

For MePa and GA3 analysis, negative polarity was used. The DP was set to -90 V for GA3 analysis and -50 V for MePa analysis. The CE and the CXP varied for each transition. Quantifier MePa (M-H)^-^: *m*/*z* 151.0→92.0 (CE -23 V, CXP -11 V), qualifier MePa: *m*/*z* 151.0→121.0 (CE -28 V, CXP -11 V). Quantifier GA3 (M-H)^-^: *m*/*z* 345.0→239.1 (CE - V, CXP -19 V), qualifier 1 GA3: *m*/*z* 345.0→143.0 (CE -36 V, CXP -13 V), qualifier 2 GA3: *m*/*z* 345.0→221.0 (CE -28 V, CXP -11 V). All experiments were performed in triplicates and a Student’s *t*-test was done to determine statistical significance.

To quantify SMs *in planta*, 20 mg of ground rice plants in case of *F. fujikuroi* and 100 mg of ground wheat ears in case of *F. graminearum* were extracted as described in Bueschl et al. (2014). Briefly, samples were extracted with 1 mL extraction solution containing methanol (1% FA) and 1% FA (3:1, v/v) and then treated with ultrasound for an additional period of 15 min. The extract was centrifuged for 10 min at 20817 × *g* and 4°C. An aliquot of 700 μL was transferred to a clean tube. After addition of 350 μL 1% FA and agitation, samples were directly used for HPLC–MS/MS measurements as previously described (Malachová et al., 2014). Mock-infected rice and wheat plants served as negative controls. Quantified metabolite levels were normalized to the input sample weights. For metabolite quantification, the concentration of the desired SMs was related to the biomass accumulation; production level of the wild type was arbitrarily set to 100% for direct comparison.

### Chromatin Immunoprecipitation (ChIP)

*F. fujikuroi* strains were grown for 72 h in DVK medium at 28°C. An aliquot of 0.5 mL was then transferred to synthetic ICI medium with either 6 or 60 mM glutamine as the sole nitrogen source. After an additional growth period of 72 h, the mycelium was crosslinked with 1% (v/v) formaldehyde for 15 min at 28°C and 90 rpm. In case of *F. graminearum*, the fungal strains were grown on solid PDA at 20°C and harvested 4 days post-inoculation. For crosslinking, mycelia were transferred to liquid potato dextrose broth containing 1% (v/v) formaldehyde and incubated for 15 min at 20°C and 90 rpm. Quenching was performed in both cases by addition of 125 mM glycine. Mycelia were filtered over miracloth and directly ground in liquid nitrogen. The following antibodies were used in this study: H3K4me2 (Active Motif, AM39141), H3K4me3 (Merck Millipore MP#07-473) and H3K9ac (Active Motif AM39137). The ChIP protocol was adapted from the protocol described elsewhere (Reyes-Dominguez et al., 2012). Sonication was performed in a Bioruptor^®^ Standard sonication device (Diagenode) for 30 min, 2 min on/1 min off at maximum power. Precipitation of the protein-antibody conjugate was performed with Dynabeads^®^ Protein A (Novex^®^, Life Technologies, 10002D). Washing was done applying 3x Wash Buffer and 1x Final Wash Buffer followed by elution in TES buffer (50 mM Tris-HCl pH 8, 10 mM EDTA, 1% SDS). Histone-bound DNA was treated with Proteinase K (Thermo Scientific) and DNA purification was done using the MiniElute PCR Purification Kit (Qiagen). Amplification and detection of precipitated DNA in qRT-PCR was performed with iQ^TM^ SYBR^®^ Green Supermix (Bio-Rad) following the providers’ instructions. All experiments were performed in biological and technical duplicates.

### Reverse Transcriptase-Quantitative PCR (RT-qPCR) and ChIP-coupled qPCR

Reverse transcriptase-quantitative PCR (RT-qPCR) was performed with iQ SYBR Green Supermix (Bio-Rad, Munchen, Germany) using an iCycler iQ Real-Time PCR System (Bio-Rad). To quantify mRNA levels of SM genes, the following primers were used: AUR gene cluster (*AUR1*, Aur1_ORF_fwd//Aur1_ORF_rev; *PKS12*, RT-PCR_PKS12_F//RT-PCR_PKS12_R), DON gene cluster (*TRI5*, Tri5_ORF_fwd//Tri5_ORF_rev; *TRI6*, Tri6_ORF_fwd //Tri6_ORF_rev), ZON gene cluster (*PKS13*, RT-PCR_PKS13_F//RT-PCR_PKS13_R; *PKS4*, PKS4-PS.2//PKS4-PS.1). Levels of mRNA were related to constitutively expressed reference genes, i.e., *FGSG_06257* encoding glyceraldehyde 3-phosphate dehydrogenase (GAPDH) (GAPDH_qPCR_fwd//GAPDH_qPCR_rev), *FGSG_07335* encoding actin (qPCR_actin_F// qPCR_actin_R) and *FGSG_09530* encoding ß-tubulin (cDNA_ß-TUB_F //cDNA_ß-TUB_R). Primer efficiencies in the RT-qPCR were kept between 90 and 110%. Relative expression levels were calculated using the △△Ct method (Pfaﬄ, 2001). Experiments were performed in biological and technical duplicates. Primer sequences are listed in Supplementary Table S1.

To reveal changes in the desired histone marks at SM cluster genes, the following primer pairs were used for qPCR in case of *F. fujikuroi*: copalyl diphosphate synthase/kaurene synthase */KS* (*FFUJ_14336*, qRT-PCR_CPS/KS_F//qRT-PCR_CPS/KS_R), P450 monooxygenase *P450-1* (*FFUJ_14333*, qRT-PCR_P450-1_F//qRT-PCR_P450-1_R), *P450-2* (*FFUJ_14334*, qRT-PCR_P450-2_F//qRT-PCR_P450-2_R) and *P450-4* (*FFUJ_14332*, qRT-PCR_P450-4_F//qRT-PCR_P450-4_R) for the GA gene cluster, PKS *BIK1* (*FFUJ_06742*, qRT-PCR_bik1_F//qRT-PCR_bik1_R) and the Zn_2_Cys_6_ transcription factor (TF) *BIK5* (*FFUJ_06746*, qRT-PCR_bik5_F//qRT-PCR_bik5_R) in case of the BIK gene cluster, PKS – NRPS *FUS1* (*FFUJ_10058*, qRT-PCR_fus1_F//qRT-PCR_fus1_R) and a pathway-specific methyltransferase *FUS9* (*FFUJ_10050*, qRT-PCR_fus9_F//qRT-PCR_fus9_R) for the FUS gene cluster. Sequences of the respective ORFs were extracted from the publicly available genome sequence of *F. fujikuroi* (Wiemann et al., 2013). In case of *F. graminearum*, the GAL4-like Zn_2_Cys_6_ TF *AUR1* (*FGSG_02320*) and *PKS12* (*FGSG_02324*) (Frandsen et al., 2006) were analyzed with primers Aur1_K4me_fwd and Aur1_K4me_rev as well as Pks12_K4me_fwd and Pks12_ORF_rev, respectively. In case of the core DON gene cluster (Desjardins et al., 1993; Hohn et al., 1993; Kimura et al., 2003), the trichodiene synthase (sesquiterpene cyclase) gene *TRI5* (*FGSG_03537*) (Hohn and Beremand, 1989) was analyzed with primers Tri5_K4me_fwd and Tri5_K4me_rev; and the TF-encoding *TRI6* gene (*FGSG_16251*) (Proctor et al., 1995; Hohn et al., 1999) was analyzed with primers Tri6_K4me_fwd and Tri6_K4me_rev. Relative amounts of DNA were calculated by dividing the immune-precipitated DNA by the input DNA. Experiments were performed in duplicates and each ChIP was done in two biological repetitions.

For determining the *F. fujikuroi* and *F. graminearum* infection rates, total gDNA (of the fungus and the plant) was isolated from infected and lyophilized plant material using the NucleoSpin Plant II Kit (Machery Nagel) for the *F. fujikuroi* infection as well as the DNeasy^®^ Plant Mini Kit (Qiagen) for the *F. graminearum* infection. Calculation of the infection rate is based on qPCR quantification of the proportion of fungal gDNA within the fungus/plant gDNA mixture (Brunner et al., 2009). Subsequent qPCR analysis was performed using iQ^TM^ SYBR^®^ Green Supermix (Bio-Rad) and primers BIK1_F//BIK1_R for quantification *F. fujikuroi* gDNA, primers Pks12_ORF_fwd//Pks12_ORF_rev for quantification of *F. graminearum* gDNA as well as primers ITS1P//ITS4 for quantification of plant gDNA (wheat or rice).

## Results

### Identification and Cloning of *CCL1* in *F. fujikuroi* and *F. graminearum*

The *cclA* homologs in *F. fujikuroi* and *F. graminearum*, *FfCCL1 and FgCCL1*, respectively, were identified by BLASTp analysis using the protein sequence of *Aspergillus nidulans* (AN9399). Clustal W alignment showed 83.6% sequence identity of Ccl1 on protein level in both fusaria (Supplementary Figure S4). To study the functions of *FfCCL1* (*FFUJ_04433*) and *FgCCL1* (*FGSG_09564*), both genes were deleted by homologous integration of a hygromycin resistance cassette into the respective wild type strains IMI58289 (*F. fujikuroi*) and PH-1 (*F. graminearum*), hereafter referred to as FfWT and FgWT. Correct integration of the resistance cassette was subsequently verified by diagnostic PCR and Southern blot analysis (Supplementary Figures S2B,C). Three independent deletion mutants each, that is Δ*ffccl1*_T9, Δ*ffccl1*_T10 and Δ*ffccl1*_T11 as well as Δ*fgccl1*_T1, Δ*fgccl1*_T3 and Δ*fgccl1*_T4, were obtained for *F. fujikuroi* and *F. graminearum*, respectively. All mutants of *F. fujikuroi* and *F. graminearum* showed an identical phenotype. Hence, Δ*ffccl1*_T9 and Δ*fgccl1*_T1 were arbitrarily chosen for complementation approaches. Complementation was done by homologous re-integration of the native genes driven by the native promoters into the Δ*ffccl1*- and Δ*fgccl1*-mutant backgrounds resulting in three independent mutants each for *F. fujikuroi* (FfCcl1^Cil^_T2, T7, T14) and *F. graminearum* (FgCcl1^Cil^_T6, T7, T8), that showed identical phenotypes and correct *in loco* integration of *FfCCL1* and *FgCCL1*, respectively (Supplementary Figure S3A).

### Deletion of *CCL1* has Only Minor Impact on Fungal Development

**FIGURE 1:**
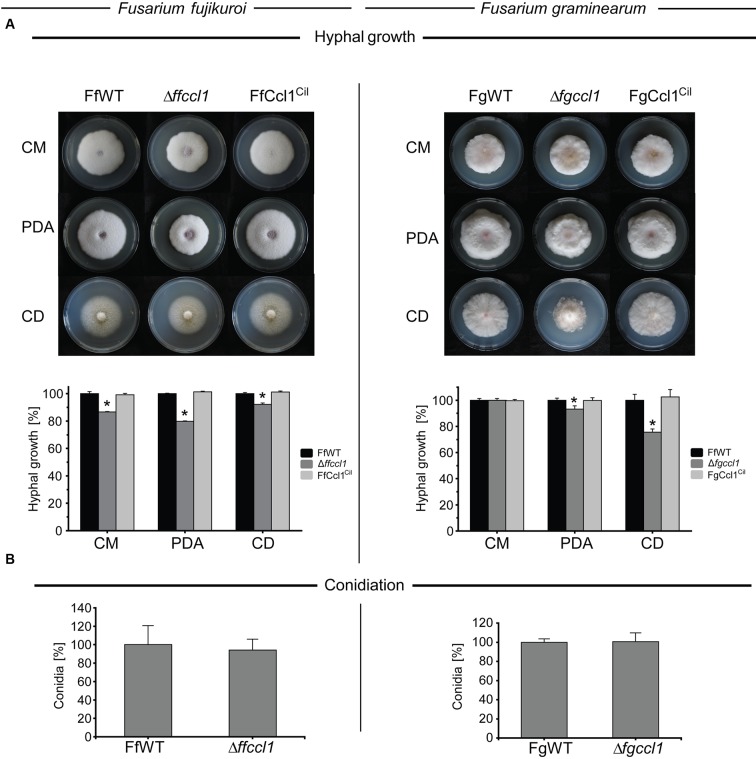
**Lack of Ccl1 results in slightly reduced hyphal growth and has no impact an asexual development. (A)** The wild type strains (*Fusarium fujikuroi* – FfWT, *Fusarium graminearum* – FgWT), *CCL1* deletion mutants (*F. fujikuroi* – △*ffccl1*, *F. graminearum* – △*fgccl1*) and *in loco* complementation strains (*F. fujikuroi* – FfCcl1^Cil^, *F. graminearum* – FgCcl^Cil^) were grown for 5 days on solid complete medium (CM), PDA and Czapek Dox (CD) medium at 28 and 20°C in case of *F. fujikuroi* (left panel) and *F. graminearum* (right panel), respectively. Experiments were done in triplicates. Mean values and standard deviations are given in the diagram. Hyphal growth of the wild type strains on the respective media was arbitrarily set to 100%. Asterisks above the bars denote significant differences in the measurements of the indicated strains compared to the respective wild type, *p* < 0.001. **(B)** Conidiation was assessed on vegetable V8 agar in case of *F. fujikuroi* (left panel) and in case of *F. graminearum* in liquid carboxymethyl cellulose (right panel). Conidia production of the respective wild type strains was arbitrarily set to 100%. Experiments were done in triplicates. Mean values and standard deviations are shown. All experiments were performed with three independent *CCL1* deletion and complementation strains all showing an identical phenotype. Hence, only one of each is shown.

To determine the influence of Ccl1 on fungal development, hyphal growth and conidiation were investigated in both fungi. Hyphal growth was only slightly, but significantly, decreased in the Δ*ffccl1* deletion mutant on all selected media, i.e., CM (to about 87%), PDA (to about 80%) and Czapek Dox (CD, to about 92%), compared to FfWT (**Figure 1A**). Similarly, deletion of *FgCCL1* in *F. graminearum* resulted in slightly reduced growth on PDA (to about 93%) and CD (to about 75%) compared to FgWT, while no growth defect was observed on CM (**Figure 1A**). Complementation of △*ffccl1* and △*fgccl1* with the native genes *FfCCL1* and *FgCCl1*, respectively, rescued the wild type phenotypes (**Figure 1A**). Regarding conidia formation, there is a significant difference between both fusaria. In general, *F. fujikuroi* is able to produce micro- as well as macroconidia. However, the FfWT strain (*F. fujikuroi* IMI58289) used in this study produces only microconidia in long chains (Wiemann et al., 2013). In contrast, *F. graminearum* does not produce microconidia but only slender macroconidia with 5 to 6 septa (Leslie and Summerell, 2007). Neither micro- nor macroconidia formation was affected by deletion of *CCL1* in *F. fujikuroi* and *F. graminearum*, respectively (**Figure 1B**). Taken together, growth behavior was only slightly affected upon deletion of *CCL1* in both fungi, while conidiation remained unchanged.

### Reduced Virulence Factor Biosynthesis of Mutants in Axenic Cultures is Rescued during Pathogenesis

**FIGURE 2:**
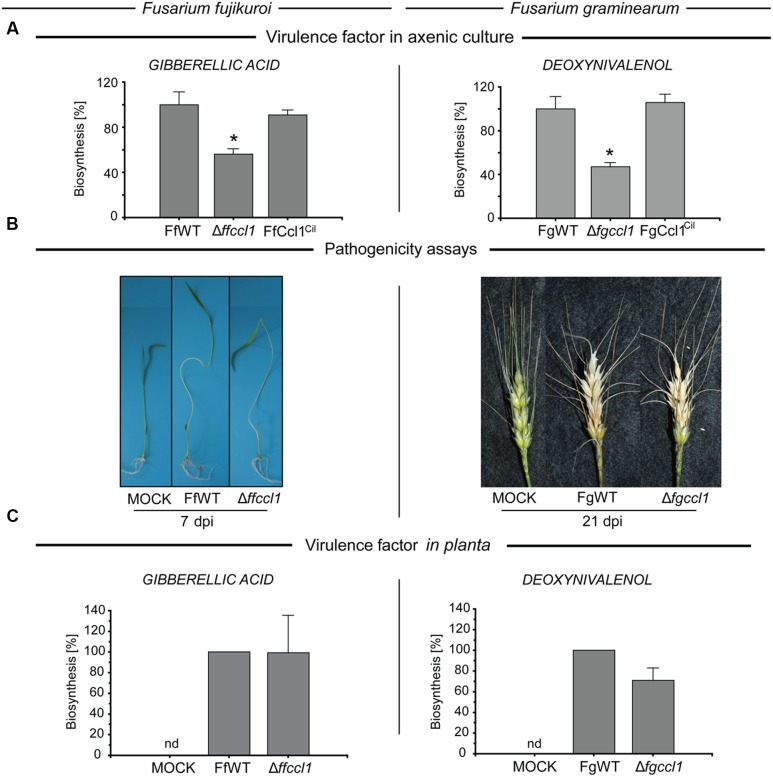
**Wild type-like virulence of △*ffccl1* and △*fgccl1* mutants is accompanied by enhanced virulence factor production *in planta* compared to the axenic cultures. (A)** The *F. fujikuroi* wild type (FfWT), △*ffccl1* and FfCcl1^Cil^ (left panel) were grown in liquid ICI medium with 6 mM glutamine for determination of gibberellic acid (GA3) at 28°C. Filtrates of 7 day-old liquid cultures were applied for HPLC–MS/MS measurements. In case of *F. graminearum* (right panel), the wild type (FgWT), Δ*fgccl1* and FgCcl1^Cil^ were grown for 2 weeks on PDA at 20°C for deoxynivalenol (DON) quantification. Agar plugs were subsequently extracted and analyzed by HPLC–MS/MS. For a direct comparison, production of the wild type was arbitrarily set to 100%. To exclude differences in the biosynthesis due to different growth behaviors, product formation was correlated to biomass formation. Experiments were performed in triplicates. Asterisks above the bars denote statistical significance, *p* < 0.005. **(B)** Rice seedlings (left panel) and wheat heads (right panel) were infected with *F. fujikuroi* FfWT and △*ffccl1* mutant and *F. graminearum* FgWT and △*fgccl1* mutant. Typical disease symptoms were assessed 7 and 21 days past infection (dpi) in case of *F. fujikuroi* and *F. graminearum*, respectively. **(C)** For *in planta* GA and DON quantification, five plants were infected each with either FfWT and △*ffccl1* or FgWT and △*fgccl1*. Plants were combined and the total amount of GA3 and DON was extracted and quantified by HPLC–MS/MS. Experiments were performed in biological duplicates; nd, not detectable.

Several SMs have been described as virulence factors, including GA and DON production in *F. fujikuroi* and *F. graminearum*, respectively (Jansen et al., 2005; Maier et al., 2006; Wiemann et al., 2013). To analyze if the deletion of *CCL1* affects the biosynthesis of the two species-specific virulence factors, the fungal wild type and mutant strains were grown under their respective production conditions, i.e., liquid synthetic ICI medium with 6 mM glutamine (nitrogen limitation) in case of GA, and PDA in case of DON (Giese et al., 2013; Wiemann et al., 2013). Deletion of *CCL1* resulted in a significantly decreased biosynthesis of both virulence factors, GA (about 56% of FfWT) in *F. fujikuroi* and DON (about 47% of FgWT) in *F. graminearum*. Complementation of the Δ*ccl1* mutant strains restored the biosynthesis of both virulence factors to the respective wild type levels (**Figure 2A**). Hence, a reduced virulence of the *CCL1* deletion mutants was expected for both fungi. However, both the *F. fujikuroi* wild type and Δ*ffccl1* mutant strains caused typical *bakanae* symptoms on rice seedlings (**Figure 2B**). No significant differences regarding shoot elongation and chlorosis compared to FfWT were observed. Similarly, infection of wheat heads with the *F. graminearum* Δ*fgccl1* mutant resulted in a phenotype that was undistinguishable from the wild type strain FgWT (**Figure 2B**). Both mutant strains showed wild type-like fungal invasion (Supplementary Figure S5). To understand the correlation between reduced production of virulence factors in axenic culture and the wild type-like virulence of both mutant strains on their respective host plants, production of GA and DON was determined also *in planta*. Unexpectedly but in agreement with the wild type-like virulence, GA and DON levels were restored to wild type levels in both mutant strains *in planta* (**Figure 2C**).

### Ccl1 Function Generates Distinct Transcriptional Readouts Depending on the SM Locus

**FIGURE 3:**
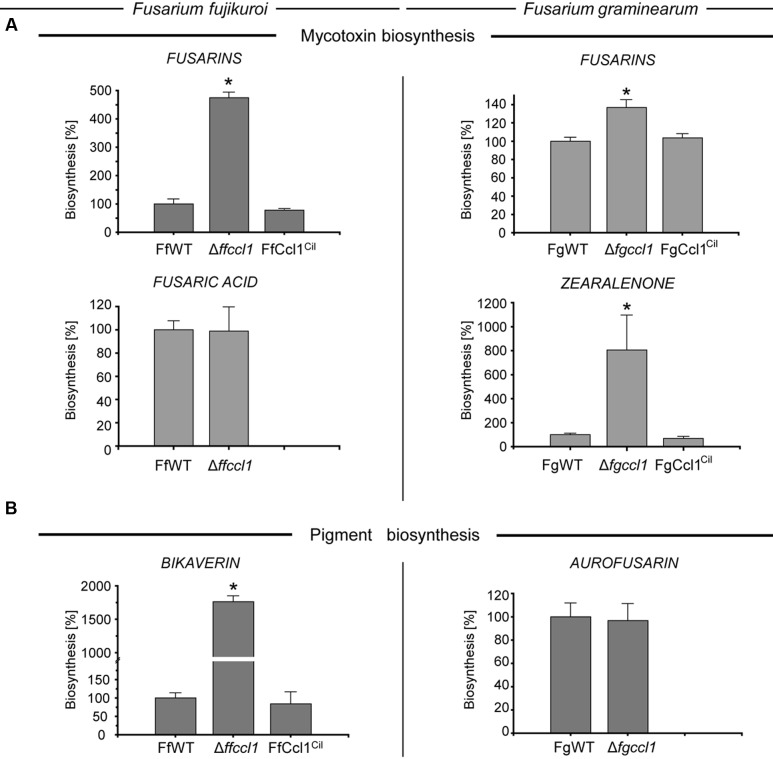
**Deletion of *CCL1* results in increased production of some secondary metabolites (SMs) in both fusaria.** The *F. fujikuroi* wild type strain (FfWT), the *CCL1* deletion mutant (△*ffccl1*) and *in loco* complementation (FfCcl1^Cil^) were grown for 7 days in synthetic ICI medium with either **(A)** 60 mM glutamine (nitrogen surplus) for fusarin and fusaric acid production or **(B)** 6 mM glutamine (nitrogen starvation) for bikaverin production (left panel). Liquid cultures were filtered over miracloth and subsequently directly applied for HPLC–DAD analysis (bikaverin) or HPLC–MS/MS (fusarins and fusaric acid). To exclude falsifications in SM biosynthesis due to differences in hyphal growth, SM production was correlated to biomass formation. The *F. graminearum* wild type strain (FgWT), the △*fgccl1* and the *in loco* complementation FgCcl1^Cil^ were grown at 20°C for 2 weeks on solid PDA for **(A)** fusarin, zearalenone and **(B)** aurofusarin production (right panel). Agar plugs were subsequently extracted and applied for HPLC–MS/MS analysis. In each case, three independent mutants were analyzed and all behaved identically, thus only one is shown. SM production of the respective wild type was arbitrarily set to 100%. Mean values and standard deviations are given. Asterisks above the bars denote significant differences in the measurements of the indicated strains compared to the respective wild type. *p* < 0.001.

To study the role of Ccl1 for the regulation of other SMs, the production of several known metabolites was analyzed in both fusaria. In *F. fujikuroi*, the production of the mycotoxins FUS and FSA, both induced under nitrogen sufficient (60 mM glutamine) conditions (Niehaus et al., 2013, 2014b), as well as production of BIK, induced under nitrogen limiting (6 mM glutamine) conditions (Wiemann et al., 2009), was assessed. In case of *F. graminearum*, production of the mycotoxins FUS and ZON as well as the pigment AUR was determined on PDA as described by Giese et al. (2013). Production yields of SMs were subsequently analyzed by HPLC or HPLC–MS/MS (Malachová et al., 2014; Michielse et al., 2014). In contrast to the reduced GA and DON levels, several other SMs were either not significantly altered (e.g., FSA in *F. fujikuroi* or AUR in *F. graminearum*) or even increased in the *CCL1* deletion strains compared to their respective wild types (**Figures 3A,B**). We noted a very strong overproduction of ZON in the *F. graminearum* mutant (about eightfold) as well as of BIK (about 18-fold) and FUS in *F. fujikuroi* (roughly 4.5-fold). Fusarins are also slightly increased (about 1.4-fold) in the *F. graminearum* mutant. Complementation of both mutant strains restored in each case the respective wild type phenotype.

### Cross-Complementation Demonstrates that Ccl1 Function is Conserved in Both Fusaria

**FIGURE 4:**
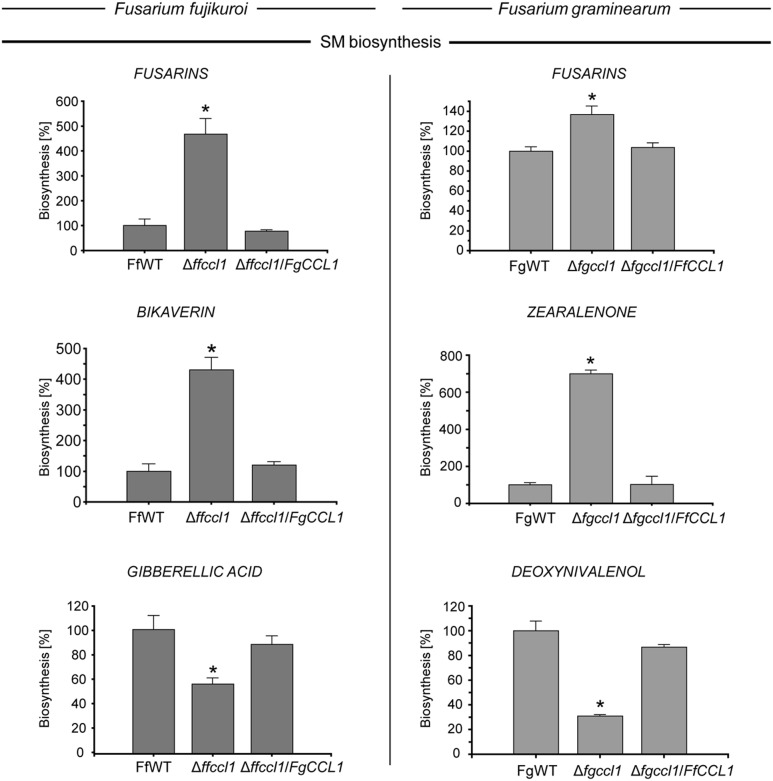
**Ccl1 shows high functional conservation in both fusaria.** For secondary metabolite (SM) production in case of *Fusarium fujikuroi* **(Left)**, the *F. fujikuroi* wild type (FfWT), *CCL1* deletion (△*ffccl1*) and cross-complementation strains (△*ffccl1/FgCCL1*) were grown in liquid synthetic ICI medium with either 60 mM (fusarin) or 6 mM (bikaverin and gibberellic acid) glutamine at 28°C. Liquid cultures were filtered over miracloth and directly used for HPLC–DAD (in case of bikaverin) or HPLC–MS/MS (in case of fusarins and gibberellic acid) analysis. To exclude differences in the biosynthesis due to different growth behaviors, product formation was correlated to the biomass. In case of *Fusarium graminearum* **(Right)**, the wild type (FgWT), △*fgccl1* and △*fgccl1/FfCCL1* strains were cultivated for 2 weeks on PDA at 20°C. Subsequently, agar plugs were extracted and applied for HPLC–MS/MS analysis. Experiments were performed in triplicates with at least three independent mutants. SM production of the respective wild type strains was arbitrarily set to 100%. Mean values and standard deviations are given. Asterisks above the bars denote significant differences in the measurements of the indicated strains compared to the respective wild type. *p* < 0.005.

To analyze if Ccl1 from one fungus is able to complement loss of the respective gene in the other, cross-complementation experiments were performed by *in loco* integration of *FgCCL1* into Δ*ffccl1* driven by the native *F. fujikuroi* promoter as well as *in loco* integration of *FfCCL1* driven by the native *F. graminearum* promoter into the Δ*fgccl1* background. Several mutants were obtained that showed correct *in loco* integration of *FgCCl1* and *FfCCL1* in the Δ*ffccl1* and the Δ*fgccl1* mutants, respectively (Supplementary Figure S3B). In both cases, three independent cross-complementation mutants were taken for subsequent experiments. As all of them showed an identical phenotype, only one of each strain, that is Δ*ffccl1/FgCCL1*_T4 and Δ*fgccl1/FfCCL1*_T22, is shown. To study if *FgCCl1* complements Δ*ffccl1*, and thus, restores SM production to FfWT-level, fungal strains were cultivated under inducing conditions for each SM (liquid synthetic ICI medium with 60 mM glutamine in case of FUS and 6 mM glutamine in case of BIK and GA). SM production was quantified after 7 days of incubation. The significantly deregulated SMs in the △*ffccl1* mutant, that is increased FUS and BIK biosynthesis and decreased GA biosynthesis, were restored to FfWT-level in Δ*ffccl1/FgCCL1* (**Figure 4**). In case of *F. graminearum*, SM production of the FgWT, △*fgccl1* and the cross-complementation strains (Δ*fgccl1/FfCCL1*) was quantified after 2 weeks of fungal growth on PDA. Similar to *F. fujikuroi*, the deregulated production of SMs in the △*fgccl1* mutant, that is increased FUS and ZON biosynthesis and decreased DON biosynthesis, was restored to FgWT-level in Δ*fgccl1/FfCCL1* (**Figure 4**). Taken together, all observed *CCL1* deletion phenotypes were restored to the respective wild type levels by cross-complementation indicating high functional conservation of Ccl1 in both distantly related fusaria.

### Ccl1 is Necessary for Full Global H3K4 Trimethylation

**FIGURE 5:**
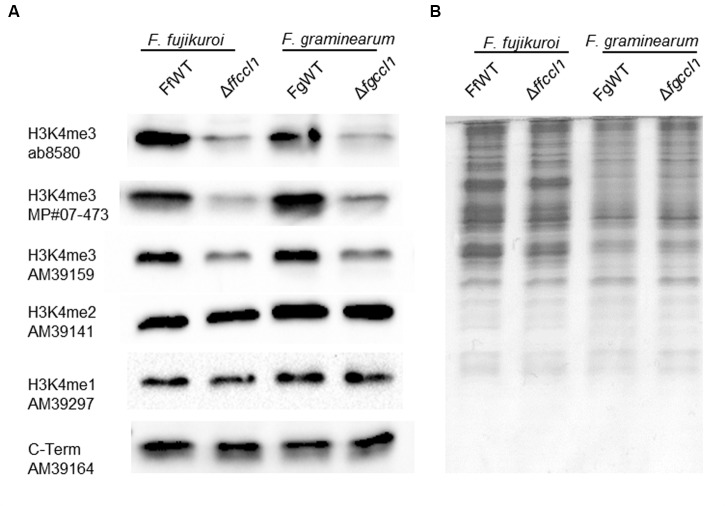
**Lack of Ccl1 results in decreased global H3K4me3.** The *Fusarium* wild type strains (*F. fujikuroi* – FfWT, *F. graminearum* – FgWT) and the respective *CCL1* deletion mutants (*F. fujikuroi* – Δ*ffccl1*, *F. graminearum* – Δ*fgccl1*) were grown for 3 days on solid complete medium. Whole protein extracts were subsequently isolated from lyophilized mycelia and roughly 15 μg of proteins were used for SDS-Page and western blotting. **(A)** The following primary antibodies were used for detection: H3 C-Term, H3K4me1, H3K4me2 as well as three different H3K4me3. **(B)** Coomassie staining was performed as an additional loading control.

To analyze the impact of *CCL1* deletion on H3K4 methylation of all nuclear histones in both fusaria, we performed western blot analysis using H3K4 mono-, di-, and trimethylation-specific antibodies (**Figure 5**). In both fungi, deletion of *CCL1* resulted in strongly reduced, but not abolished, H3K4me3-marked histones. Because antibody specificity is always an issue in chromatin analysis, we used different antibody sources for H3K4me3 and all gave comparable results. Complementation as well as cross-complementation restored H3K4me3 to wild type levels in both fungi (Supplementary Figure S6). Thus, similar to the findings in *S. pombe*, *Arabidopsis thaliana*, and *Drosophila melanogaster* (Jiang et al., 2011; Mohan et al., 2011; Mikheyeva et al., 2014), lack of the bre2 homolog Ccl1 causes great decrease in genome-wide H3K4me3 but not in H3K4me1/2 levels in both *Fusarium* spp.

### Ccl1 Regulates Balance between H3K4 Di- and Trimethylation Independently of Transcriptional Status of SM Genes

Next we analyzed H3K4 methylation levels in more detail at the affected SM clusters using these antibodies to analyze gene-specific H3K4 methylation by qPCR-coupled ChIP. To identify possible gene-specific changes in the *CCL1* deletion mutants, we tested genes in the FUS, BIK and GA clusters in *F. fujikuroi* as well as genes in the FUS, ZON and DON clusters in *F. graminearum.* For this, *F. fujikuroi* strains were cultivated in synthetic ICI medium under nitrogen starvation (6 mM glutamine for BIK and GA) and nitrogen surplus (60 mM glutamine for FUS) conditions for 3 days, a time point when transcripts of all investigated SMs are clearly detectable in the FfWT (Wiemann et al., 2009; Niehaus et al., 2013, 2014b). In case of *F. graminearum*, ChIP was performed from cultures grown for 4 days on solid PDA, when transcripts were detectable in the *FgCCL1* deletion strain and differences in SM transcription were highest (Supplementary Figure S7). Importantly, expression of all analyzed SM biosynthetic genes is in agreement with the SM levels recorded in the wild type and mutant strains (compare **Figure 2** and Supplementary Figure S7).

**FIGURE 6:**
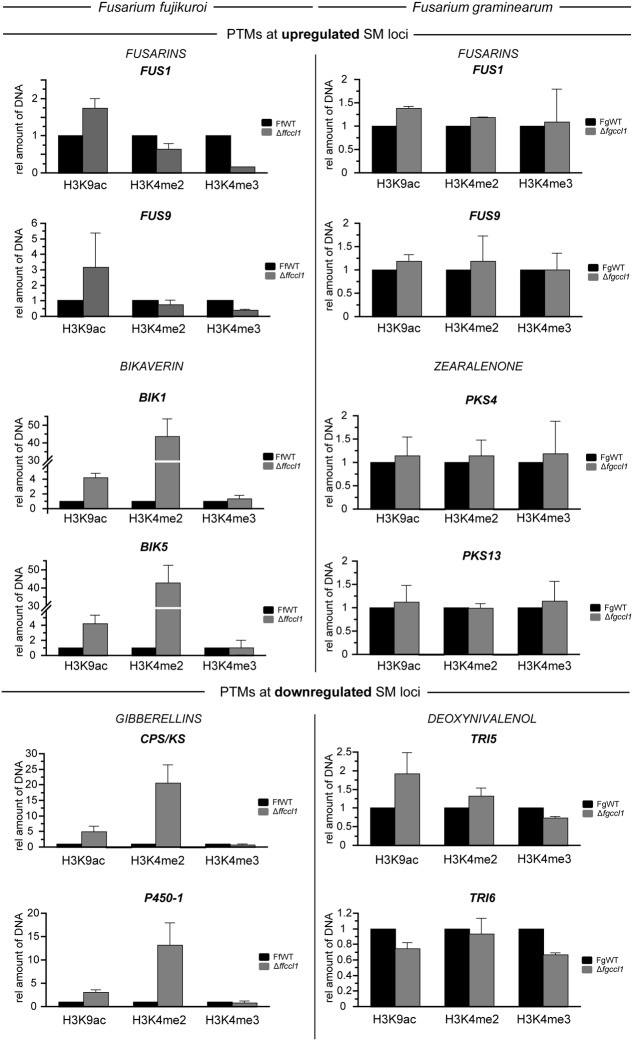
**Chromatin immunoprecipitation (ChIP) reveals distinct alterations in H3K4 dimethylation at SM gene clusters.** The *F. fujikuroi* wild type strain (FfWT) and the *CCL1* deletion mutant (Δ*ffccl1*) were grown in synthetic ICI medium with either 60 mM (fusarin) or 6 mM glutamine (bikaverin and gibberellic acid) for 3 days. Mycelium was crosslinked and used for ChIP analyses **(Left**). In case of *F. graminearum*, the wild type (FgWT) and Δ*fgccl1* were grown for 4 days on PDA **(Right**). ChIP assays were conducted by using H3K9 acetylation (H3K9ac)-, H3K4me2- and H3K4me3-specific antibodies. Precipitated genomic DNA was quantified by quantitative real-time PCR using primer pairs located in the 5′ region of investigated SM cluster genes. For each SM cluster, two cluster genes were analyzed. Experiments were performed in biological and technical duplicates. The amount of precipitated DNA in the respective wild type strain was arbitrarily set to 1. Mean values and standard deviations are shown. PTMs, post-translational modifications.

Primers for gene-specific qPCR after ChIP were designed to target in each case the first one or two nucleosomes of the coding region, because it has been shown in numerous organisms including *F. graminearum* (Connolly et al., 2013) and *A. nidulans* (Gacek-Matthews et al., 2016), that these are the ones most prominently labeled with H3K4 di- or trimethylation. Although, genome-wide reduction of H3K4me3 in Δ*fgccl1* and Δ*ffccl1* was evident in western blot analysis, no such clear changes were observed at the investigated SM cluster genes in either of the two *Fusarium* spp. (**Figure 6**), with the exception of *FUS1* in *F. fujikuroi* (**Figure 6**, left panel, first graph). Here, the relative amount of H3K4me3 in the mutant was reduced to roughly 20% compared to the FfWT. All other genes showed either no or only subtle reduction in H3K4me3 levels in the mutants consistent with the observed generally very low levels of this mark in SM gene clusters which cannot be reduced further. To validate the results from the SM gene clusters, we also quantified H3K4me3 levels in the actin-encoding gene (*ACN*) that is known to carry high levels of these marks in both fusaria. Here, in agreement with the global histone analysis, H3K4me3 levels were significantly decreased to about 53 and 54% in case of *F. fujikuroi* and *F. graminearum*, respectively (Supplementary Figure S8). At these loci, we detected for the first time an interdependency between H3K4 methylation marks as concomitantly with decreased H3K4me3 levels, the amount of H3K4me2 was increased in the *CCL1* deletion strain of *F. fujikuroi*, and slightly also in *F. graminearum*.

In the latter pathogen, the investigated SM cluster genes had no change in H3K4 dimethylation and the results were consistent in all investigated genes. In contrast, two out of three SM gene clusters in *F. fujikuroi* showed a strong switch between both H3K4me forms in the mutant. Genes in the investigated BIK as well as GA cluster showed an enormous enrichment of H3K4me2 at their 5′-end nucleosomes (**Figure 6**, left panel). It is important to note, that these two gene clusters are the ones investigated here which are positioned in typical subtelomeric regions that are known to be highly decorated with this mark and influenced by Bre2/CclA during subtelomeric gene silencing (Miller et al., 2001; Krogan et al., 2002; Bok et al., 2009; Mikheyeva et al., 2014).

In a previous study employing ChIP-seq in the FfWT, we found out of all analyzed SM gene clusters induced under nitrogen-limiting conditions significant H3K4me2 levels in only two genes of the GA cluster (*P450-2* and *P450-4*) that are (Wiemann et al., 2013). To analyze if the observed changes in dimethylation levels can also be seen at these two genes, H3K4me2 levels were additionally quantified for *P450-2* and *P450-4*. Similar to *CPS/KS* and *P450-1*, H3K4me2 levels were also significantly increased at the two *P450* genes upon deletion of *FfCCL1* (17-fold at *P450-2* and 12-fold at *P450-4*) (Supplementary Figure S9). Notably, enrichments in H3K4me2 were comparable to H3K4me2 levels at *ACN* (Supplementary Figure S10). However, expression of biosynthetic activities did not necessarily correlate with this strong H3K4me2 decoration as in the case of bikaverin, the genes carrying high H3K4me2 are more active in the mutant whereas the GA cluster is less active in the mutant despite the strongly increased H3K4me2 levels.

As gene expression of several SM clusters was altered profoundly, we also examined the H3K9 acetylation status (H3K9ac) in wild type and *CCL1* mutant strains. Previously, we have shown that H3K9ac is associated with active transcription of several SM gene clusters in *F. fujikuroi*, including the ones investigated here (Niehaus et al., 2013; Studt et al., 2013). We found that H3K9ac levels were significantly increased in the Δ*ffccl1* mutant at the FUS (about 1.9-fold for *FUS1* and 4.1-fold for *FUS9*) and the BIK cluster genes (4.1-fold for *BIK1* and 4.2-fold for *BIK5*) (**Figure 6**), which is in agreement with their elevated expression activities. However, also the GA cluster showed significant enrichment of H3K9ac in the *FfCCL1* deletion strain (fivefold at *CPS/KS*, threefold at *P450-1*, threefold compared to FfWT) (**Figure 6**), albeit a reduced GA production level was recorded under these conditions (**Figure 3A**). In case of *F. graminearum*, a slight yet statistically significant increase in H3K9ac was seen only for some of the investigated SM genes upon deletion of *CCL1*, but it is questionable if these small effects are biologically relevant.

## Discussion

In close proximity to promoters, H3K4me3 is thought to promote the transition of RNA polymerase II (Pol II) to active elongation (Ardehali et al., 2011). Methylation of H3K4 is mediated by the methyltransferase Set1 that catalyzes mono-, di- and trimethylation and is itself integrated in the COMPASS complex (Roguev et al., 2001; Krogan et al., 2002; Schneider et al., 2005). In contrast to animals and yeasts, where the roles of Set1 and its complex partners are firmly established in transcriptional control and genome stability (Shilatifard, 2012), the function of Set1 was studied just in a few cases in the filamentous ascomycetes (pezizomycotina). In *Neurospora crassa* Set1 is not essential and has been shown to regulate the circadian rhythm (Raduwan et al., 2013). Also in *A. nidulans set1*Δ alone is viable but synthetic lethal with regulators of mitosis which provides a link between H3K4 methylation and H3S10 phosphorylation (Govindaraghavan et al., 2014). Cells of the rice pathogen *M. oryzae* lacking MoSet1 cannot develop proper infection and asexual reproduction structures (Pham et al., 2015). In contrast to the quite mild phenotypes of Set1 inactivation mutants in the species mentioned above, lack of this gene resulted in severe growth defects, increased stress sensitivity and reduced virulence in *F. graminearum* (Liu et al., 2015) as well as crippled growth and an almost abolished asexual reproduction in *F. fujikuroi*. Taken together, this indicates that H3K4 methylation is an essential chromatin modification in fusaria required for a number of cellular processes whereas in *N. crassa* and *A. nidulans* this histone mark seems to play only a role in some specialized processes.

### Ccl1 is Largely Dispensable for Normal Fungal Growth and Development

While lack of Set1, the catalytic subunit of COMPASS, has drastic effects in both fusaria, removal of the putative adaptor subunit by deletion of *CCL1* had only minor impacts on hyphal growth and conidiation in these two fungi. Western blot analysis showed that in this mutant only H3K4 trimethylation levels are reduced, whereas H3K4 mono- and dimethylation remain basically unchanged within the limits of detection by this method. This suggests that mono- and dimethylation are important features of chromatin in these two species but the observed genome-wide decrease in H3K4me3 does not drastically alter genome function and essential transcriptional networks. Again, this is contrary to findings in *Aspergillus* spp., where lack of the *CCL1* homolog *cclA* in *A. nidulans* and *Aspergillus fumigatus* resulted in reduced hyphal growth on various media (Bok et al., 2009; Giles et al., 2011; Palmer et al., 2013). A similar growth defect has been observed in *A. nidulans set1* and *swd1* deletions indicating that the integrity of the COMPASS complex is important for full vigor of this fungus. In fact, recent ChIP-seq analysis of *A. nidulans* cells also provided compelling evidence that basic metabolism genes are highly enriched for H3K4me3 (Gacek-Matthews et al., 2016), and thus loss of this mark can explain the pleiotropic effects on growth and development observed upon deletion of *cclA* in this fungus.

One possible explanation for the relatively mild effect in *CCL1* deletion strains of both fusaria would be that, in contrast to *Aspergillus* spp. (Palmer et al., 2013) mammals, and yeast (Schlichter and Cairns, 2005; Schneider et al., 2005; Dehé et al., 2006; Dou et al., 2006), deletion of *CCL1* in our *Fusarium* species did not cause any detectable changes in H3K4me2 and some H3K4me3 marks were still present. Notably, H3K4me3 levels at the actin-encoding genes were reduced though not abolished, suggesting that genes crucial for fungal growth and development keep sufficient levels of H3K4me3 even in the absence of *CCL1*.

### Ccl1 Alters Secondary Metabolism Independently of H3K4 Methylation at SM Loci

Apart from the slight differences in the methylation pattern in *F. fujikuroi* and *F. graminearum CCL1* deletion compared to *Aspergillus* spp., there is also a similarity between both genera as secondary metabolism was significantly altered upon deletion of *CCL1*. In both we find up-regulation of SMs produced by genes localized near the telomeres in the respective fungi. In these genomic regions H3K4me3 and several subunits of the COMPASS complex have been shown to mediate gene silencing although the exact mechanism of this process has not been elucidated yet (Nislow et al., 1997; Miller et al., 2001; Krogan et al., 2002; Sims et al., 2003; Schneider et al., 2005; Wood et al., 2005; Venkatasubrahmanyam et al., 2007; Bok et al., 2009; Margaritis et al., 2012). Going in line with this, lack of *cclA* resulted in the induction of monodictyphenone/emodin and the two antiosteoporosis polyketides F9775A and F9775B in *A. nidulans*, increased gliotoxin production in *A. fumigatus* as well as induction of the sesquiterpenoid astellolides in *A. oryzae* (Bok et al., 2009; Palmer et al., 2013; Shinohara et al., 2016). Strikingly, both non-reducing PKS-encoding genes, *mpdG* (AN0150) and *orsA* (AN7909), involved in the biosynthesis of monodictyphenone/emodin and F9775A/F9775B, respectively, are localized in subtelomeric regions in *A. nidulans* (Klejnstrup et al., 2012). Notably, lack of the KDM5-family histone demethylase KdmB, involved in the removal of H3K4 di- and trimethylation marks, showed an identical phenotype concerning these two polyketides (Gacek-Matthews et al., 2016), suggesting that KdmB targeting H3K4 methylation plays a role in negative regulation of these clusters in some way. Similarly, several of the confirmed and predicted SM gene clusters in *F. fujikuroi*, including GA and bikaverin, are located in subtelomeric regions (Wiemann et al., 2013). In *F. graminearum*, predicted SM gene clusters are largely localized in regions enriched for H3K27me3, including subtelomeric but also non-subtelomeric high diversity regions, that likely result from chromosome fusions reflecting ancestral telomeres and subtelomeric regions (Cuomo et al., 2007; Connolly et al., 2013). Thus, it is tempting to speculate that Ccl1 functions in silencing of SM gene expression via regulating H3K4 trimethylation also in these two fungi.

In *A. nidulans*, increase in SM production was accompanied by reduced H3K4 di- and trimethylation levels at selected monodictyphenone cluster genes and the authors concluded that high levels of these marks hinder gene expression (Bok et al., 2009). However, recent genome-wide ChIP-seq experiments of *A. nidulans* and *F. graminearum* revealed that, with few exceptions, most of the SM cluster genes are poorly decorated with these activating marks even under inducing condition (Connolly et al., 2013; Gacek-Matthews et al., 2016). In accordance with this, only few SM cluster genes harbor H3K4me2 in *F. fujikuroi*, including *P450-2* and *P450-4* of the GA cluster (Wiemann et al., 2013), and unchanged H3K4me3 levels at the investigated SM gene clusters in the Δ*ccl1* mutant suggest that this mark is also not abundant at SM gene clusters in *F. fujikuroi*. Thus, it is questionable if Ccl1 functions directly in SM gene repression at subtelomeric regions by positioning H3K4 trimethylation at these loci. In fact, as long as the exact mechanism of subtelomeric gene silencing has not been deciphered, we consider it more likely that Ccl1 rather acts indirectly by regulating additional *cis*-/*trans*-acting factors on transcription of affected SM gene clusters. This interpretation is further underlined by observations from this study in which we found no direct correlation between *CCL1* disruption, H3K4me3 and the effect on transcriptional activity of SM biosynthetic genes.

Similarly, in *M. oryzae* where the methyltransferase MoSet1 plays a role in gene activation as well as gene repression, no significant changes in H3K4 di- and trimethylation at MoSet1-repressed gene groups were found (Pham et al., 2015). Thus, also in this pathogen MoSet1-dependency in the downregulated genes do not seem to be a direct consequence of changes in the H3K4 methylation pattern at the respective gene loci, but rather a result of indirect genetic events. A similar scenario can be imagined for the observed SM phenotypes in the △*ccl1* mutants, in which de-repression of SM genes and concomitant increase in SM production could be due to the insufficient activation of a repressor protein. Overall, transcriptional regulation by H3K4 methylation appears to be different between subtelomeric regions and the body of chromosomes and only future studies directly targeting the localization of COMPASS subunits by ChIP will be able to discern direct from indirect effects of regulation by these important transcriptional modulators.

### Reduced Virulence Factor Production is Rescued during Pathogenesis

A very interesting aspect of this study was the finding that pathogenic conditions could reconstitute the very low production of GA and DON in *CCL1* mutants grown under axenic conditions in shake flasks. The wild type-like virulence of the *CCL1* mutants is probably also the results of this reconstitution effect since both GA and DON are main virulence factors in *F. fujikuroi* and *F. graminearum*, respectively. These findings suggest that a so far unknown plant-derived signal can compensate for the lack of Ccl1 *in planta* and remediates virulence factor production. A similar phenotype has been observed for a mutant lacking a global activator of secondary metabolism, FfSge1, in *F. fujikuroi*. Here, GA production was nearly abolished in axenic culture, while GA production was increased up to 50% of the wild type level during pathogenesis (Michielse et al., 2014). These findings indicate that different regulatory mechanisms operate *in planta* compared to axenic cultivations, and it is tempting to hypothesize that plant-derived signals not present in axenic cultures function in the pathogen at the level of chromatin and trigger the production of SMs which are used then by the fungi to suppress plant defense.

## Author Contributions

LS, BT, and JS contributed to the design of the work; LS, SJ, BA, MS, and SB were involved in data acquisition; LS, SJ, BT, JS, BA, H-UH, and MS were involved in data analysis; LS and JS wrote the manuscript. All authors revised and approved the manuscript.

## Conflict of Interest Statement

The authors declare that the research was conducted in the absence of any commercial or financial relationships that could be construed as a potential conflict of interest.
