# Supplementary material for: Lack of the COMPASS Component Ccl1 Reduces H3K4 Trimethylation Levels and Affects Transcription of Secondary Metabolite Genes in Two Plant–Pathogenic Fusarium Species
Source: Front Microbiol. 2017 Jan 9;7:2144. doi: 10.3389/fmicb.2016.02144 (PMC5220078; doi:10.3389/fmicb.2016.02144)
Supplement: Supplementary file 1 [file Data_Sheet_1.docx]

*Supplementary Material*

**Lack of the COMPASS component Ccl1 reduces H3K4 trimethylation levels and affects transcription of secondary metabolite genes in two plant-pathogenic *Fusarium* species**

Lena Studt1,2,*, Slavica Janevska2, Birgit Arndt^3^, Stefan Boedi1, Michael Sulyok4, Hans-Ulrich Humpf3, Bettina Tudzynski2, and Joseph Strauss^1,*^

^1^Division of Microbial Genetics and Pathogen Interactions, Department of Applied Genetics and Cell Biology, BOKU-University of Natural Resources and Life Science Vienna, Campus Tulln, Austria

2Institute for Plant Biology and Biotechnology, Westfälische Wilhelms University, Münster, Germany

3Institute of Food Chemistry, Westfälische Wilhelms University, Münster, Germany

^4^Center for Analytical Chemistry, Department IFA-Tulln, BOKU-University of Natural Resources and Life Science Vienna, Campus Tulln, Austria

^*^corresponding authors

**Correspondance:**

Lena Studt

[lena.studt@boku.ac.at](mailto:lena.studt@boku.ac.at)

Joseph Strauss

Joseph.strauss@boku.ac.at

Table S1 Primers used in this study

| ***Primer ID*** | ***Primer sequence*** |
| --- | --- |
| ***Primers used for plasmid generation*** | |
| FfCCL1_5F | GTAACGCCAGGGTTTTCCCAGTCACGACGCCTTGCTTCTGCCTTGTCTTC |
| FfCCL1_5R | ATCCACTTAACGTTACTGAAATCTCCAACGACTGAGGCGTTGACGATATTG |
| FfCCL1_3F | CTCCTTCAATATCATCTTCTGTCTCCGACAGCCTCTAGAAGAGATGTTC |
| FfCCL1_3R | GCGGATAACAATTTCACACAGGAAACAGCGTAATGTTCTCGACCGCCT |
| FgCCL1_5F | GCCAGGGTTTTCCCAGTCACGACGGATAGACTACAACTCAACTGGG |
| FgCCL1_5R | GGTAAAAATGGAGTGATTGAGATGATTGGC |
| FgCCL1_3F | ATATCATCTTCTGTCTCCGACTGCGTTGGAAATGCTCTGAGAACG |
| FgCCL1_3R | GATAACAATTTCACACAGGAAACAGCCACAAGAAGCCACAGGATGG |
| Hph-F | GTCGGAGACAGAAGATGATATTGAAGGAGC |
| Hph-R | GTTGGAGATTTCAGTAACGTTAAGTGGAT |
| FfCCL1C_IL_R | CATACATCTTATCTACATACGTCAATCGTCTTGGACCACTTCC |
| BcGlu-term-F2 | GCGGCCGCTTAGCGTATGTAGATAAGATGTATG |
| Tgluc-nat1-R | CCACTTAACGTTACTGAAATCTCCAACATCTTGTTGGGGGGAAGGGGT |
| Hph-R-nat1 | GTTGGAGATTTCAGTAACGTTAAGTGGATCGTATCTTATCGAGATCCTGAACACC |
| FgCCL1_IL_F | CTCAATCACTCCATTTTTACCATGACGTCCGAGCTTGGGAGCTCC |
| FgCCL1_Tgluc_IL_R | CATACATCTTATCTACATACGTCAGTCGTCTTGTACCAATTCC |
| Geni-F | GTCGGAGACAGAAGATGATATTGAAGGAGCCAACAAAACACAGTTCCGACCAC |
| Geni-Tgluc-R | ACCCCTTCCCCCCAACAAGATATCATCATGCAACATGCATGTACTG |
| FgCCL1-FfCCL1_F | CCTCAGTCGTTCCCTTTGGTCATGGTATCCGAGTTTGGAAGCTCTCC |
| FfCCL1_5R2 | GACCAAAGGGAACGACTGAGG |
| FfCCL1-IL-F | CTCAATCACTCCATTTTTACCATGACGTCCGAGCTTGGGAGCTCC |
| FfCCL1-Tgluc-IL-R | CATACATCTTATCTACATACGTCAATCGTCTTGGACCACTTCC |
| FgCCL1-FfCCL1-5R | CAAGCTCGGACGTCATGGTAAAAATGGAGTGATTGAGATGATTGGC |
| Split-mark_hph_F | CTGCCTGAAACCGAACTGCC |
| Split-mark_hph_R | GACCAATGCGGAGCATATACG |
| genR_split_F | GGGAAGGGACTGGCTGCTATTG |
| genR_split_R | GCAATATCACGGGTAGCCAACG |
| ***Primers used for diagnostic PCR*** | |
| dia_FfCCL1_5’ | GAATCGGATCTGGGCTTGTG |
| TrpC-T | GGAATAGAGTAGATGCCGACCGG |
| dia_FfCCL11_3’ | AGATAATGTCGCGCCACCAGA |
| TrpC-P | CCTCCACTAGCTCCAGCCAAGCCC |
| dia_FgCCL1_5’ | GCAATCTCGAACCAATGGG |
| dia_FgCCL1_3’ | GTCTGAGAGTGAGTTAGAGG |
| Ffccl1_WT-F | ACTCTAGATCCAAGTCTGC |
| Ffccl1_WT-R | TCACTCACACCTTGGCGAT |
| FgCCL1_WT_F | GCACTGCAAGATCATGACCG |
| FgCCL1_WT_R | CGTAGACAATATCCTCTGC |
| FgCCL1_IL_dia | CAGTGTCTCTTTCTTGGTGC |
| Nat1_R1 | CAGTGCCTCGATGGCCTCGGCGTC |
| dia_ILC_FgCCL1_5F | GATAACGGAGATGTAGTGAGCG |
| dia_ILC_FgCCL1_5R | CAGTGTCTCTTTCTTGGTGC |
| dia_ILCC_FgxFfccl1_5R | GAACGACAGGACTGTGACGG |
| Gen-Seq3 | CAGCCGATTGTCTGTTGTGC |
| dia_ILC_fgccl1_3R | GTCTGAGAGTGAGTTAGAGG |
|  |  |
| ***Primer ID*** | ***Primer sequence*** |
| ***Primers used for ChIP-qPCR and RT-qPCR*** | |
| qRT-PCR_cps/ks_F | TCAGACGCAATGTTGATATCTCGC |
| qRT-PCR_cps/ks_R | AGGTGTGTTTCATACGTACGGC |
| qRT-PCR_P450-1_F | TGGCGAACTGTCCAACGTCC |
| qRT-PCR_P450-1_R | CTGCCATGGTGAAGCTGAGC |
| qRT-PCR_P450-2_F | TGAGACTACGGCTTCTTCC |
| qRT-PCR_P450-2_R | AGACGAATATGGCGATGTAGA |
| qRT-PCR_P450-4_F | AGCATGAACAGTACCAGCC |
| qRT-PCR_P450-4_R | AAGAGGACGATAGGACATGG |
| qRT-PCR_bik1_F | CATACACATCTGCTGAGGACGC |
| qRT-PCR_bik1_R | AGGATGCATGCAGGTCTAAACC |
| qRT-PCR_bik5_F | TCGCATTTGACTCTGCGTTGG |
| qRT-PCR_bik5_R | ACGAGCACACATCCTCTAGGG |
| qRT-PCR_fus1_F | TGAGCAGTATCGCACCAGTCG |
| qRT-PCR_fus1_R | GAGGTGCCAATGATGGCTATGGG |
| qRT-PCR_fus9_F | GCTGCATGTGAACTGTAGGCG |
| qRT-PCR_fus9_R | CTCCTTCATCGAGTTCAACGC |
| qRT-PCR_fub1_F | TCAAATCCAAAGCAAGCCAGCG |
| qRT-PCR_fub1_R | AGCGTTGTGAAAGCCATTAGCG |
| qRT-PCR_fub5_F | AGCAATCGTCTCCCATGAGCC |
| qRT-PCR_fub5_R | ACCAAGGCCATCGTGAATGGG |
| Aur1_K4me_fwd | GCGACAACTGTGCCAAGTCA |
| Aur1_K4me_rev | ATGGCAGTAAAGGGCGGAGT |
| Pks12_K4me_fwd | ACGCTTTGCCCCTCCACTGA |
| Pks12_ORF_rev | CGAGTTCCTGAATGTCGGCAAA |
| Tri5_K4me_fwd | CACTAGCGTGCGCCTTCTCG |
| Tri5_K4me_rev | TTGGAGGGAAGCCTGTAGTCG |
| Tri6_K4me_fwd | CGAATCTTGGAGCGCCTTGC |
| Tri6_K4me_rev | TGGTTGAATCCACCGTTGGTAG |
| Aur1_ORF_fwd | GCTGGCGACAGCAATATCAGC |
| Aur1_ORF_rev | AGCCTCGCAGTGCTGCTTGT |
| RT-PCR_PKS12_F | GCCGCAGACATACATGCTGTGGG |
| RT-PCR_PKS12_R | CGCCTCCCAACCATTCGGACC |
| Tri5_ORF_fwd | GGAGAAGCTCACCCAGGAAACC |
| Tri5_ORF_rev | AATCTCATGGAGGCGGTATCGAG |
| Tri6_ORF_fwd | GACTTCGCGAACCCGGCTAT |
| Tri6_ORF_rev | CGTCCGCTTTCAAAGACTGTGG |
| RT-PCR_PKS13_F | CCGTTGCTACGTACCATCCACAGCC |
| RT-PCR_PKS13_R | TTGGGATGCCTCGCTTAGCCAGC |
| PKS4-PS.2 | ATGCCCTGATGAAGAGTTTGAT |
| PKS4-PS.1 | GTGGGCTTCGCTAGACCGTGAGTT |
| RT-PCR_FUS1_F | GTTCCTGTTGAAGATGTGGCGCAGGG |
| RT-PCR_FUS1_R | CGAGTCAACCACTCCTCCACGCC |
| RT-PCR_FUS9_F | CGACATCACCCATCCAGCCATCC |
| RT-PCR_FUS9_R | GCCCTCCATTGCCAACTTTGAGGG |
| **GAPDH_qPCR_fwd** | **CGTCAACGGCAAGACCATCAAGTT** |
| **GAPDH_qPCR_rev** | **CCCTTCTCGAGGCGAACAGTCAA** |
| qPCR_actin_F | TGGTGGTACCACCATGTACCCCG |
| qPCR_actin_R | GGGAAGCGAGAATGGAACCACCG |
| cDNA_ß-TUB_F | TGCGCTATTCCCCCTCGTGG |
| cDNA_ß-TUB_R | TGGACTCGGCCTCAGTGAACTCC |
| ***Primers used for detection of fungal/plant genomic DNA*** | |
| Pks12_ORF_fwd | AGATGCACGGCACAGGCACT |
| Pks12_ORF_rev | ATTCACCGTGCCCGACATTG |
| ITS1P | AACCTTATCATTTAGAGGAAGG |
| ITS4 | TCCTCCGCTTATTGATATGC |
| BIK1_F | GGCATTCTCAATCGCCATTGTGC |
| BIK1_R | CCGTCTCGTCTGTGAACGAGCG |

Fig. S1


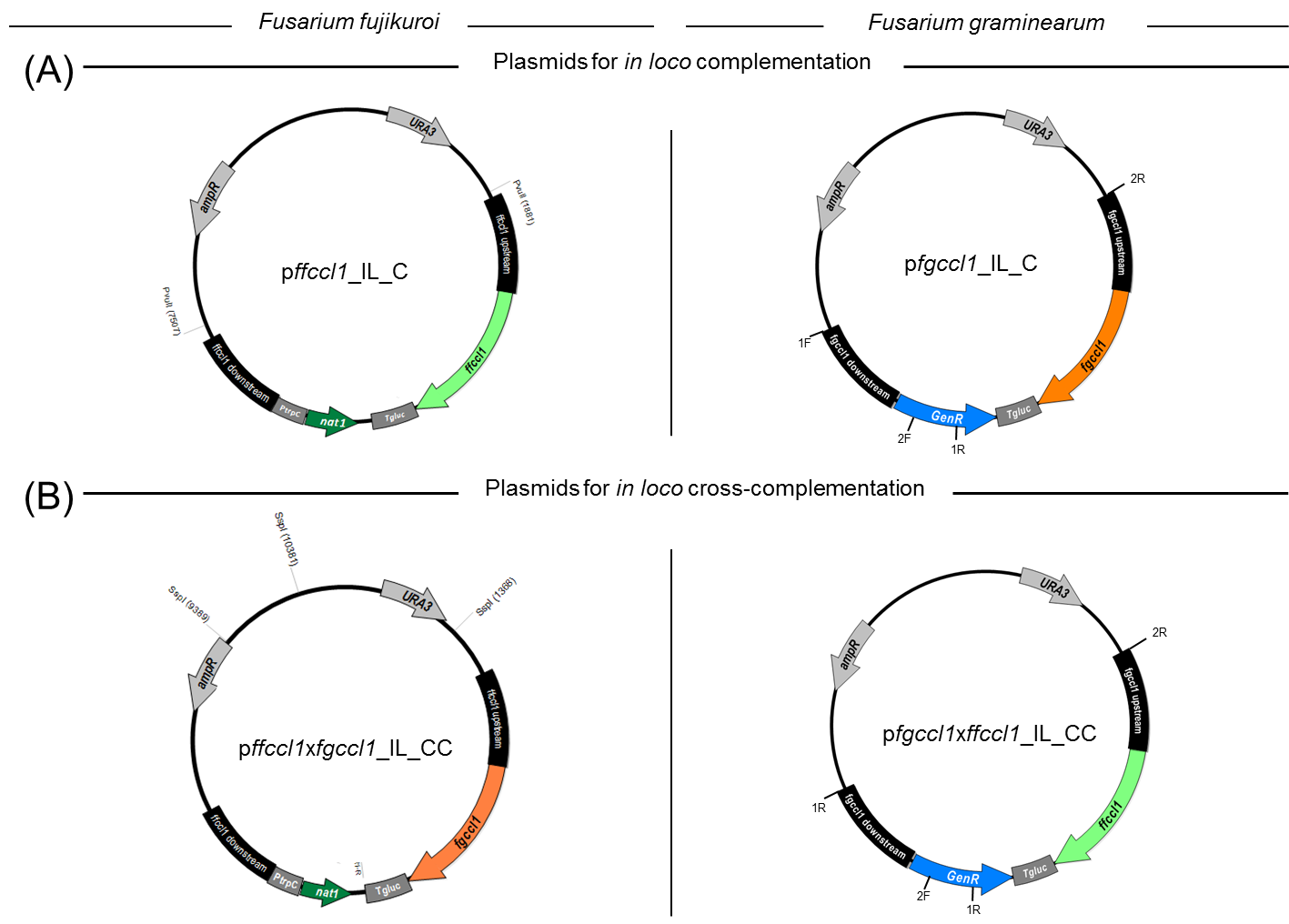


**Fig. S1 Plasmids for *in loco* complementation and cross-complementation experiments.** In case of *F. fujikuroi*, the respective enzyme cutting sites are highlighted (left panel). For *F. graminearum* primers used for amplification (1F//1R and 2F//2R) of plasmid fragments are indicated (right panel).

Fig. S2


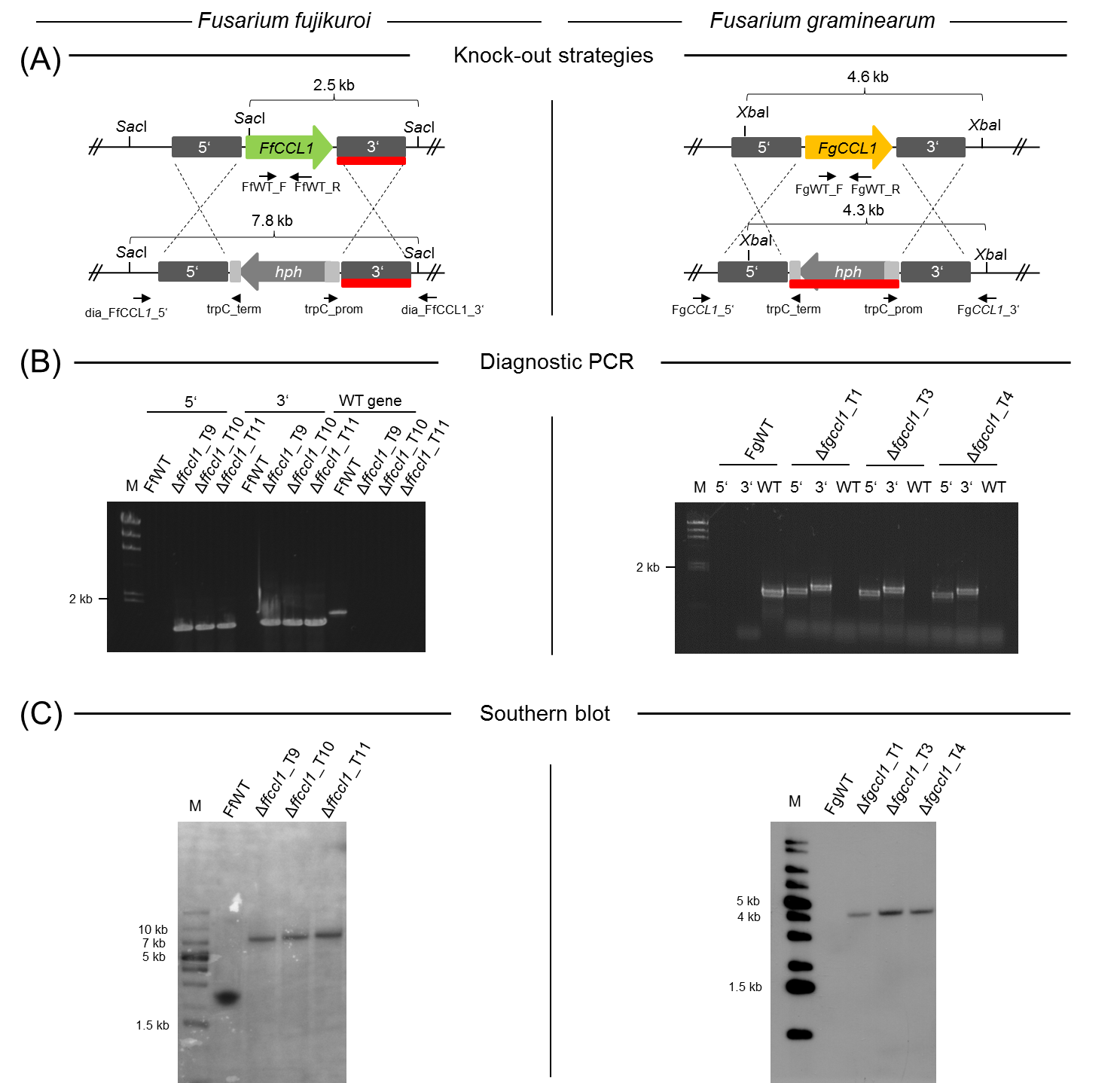


**Fig. S2 Gene replacement of *CCL1* in *F. fujikuroi* and *F. graminearum*.** A) Targeted deletion strategies for *CCL1*-gene replacement in *F. fujikuroi* (on the left) and *F. graminearum* (on the right). Primers used for diagnostic PCR are highlighted. Enzymes applied for Southern blot and respective cutting sites as well as expected fragment sizes are shown in the figure. Probes used for hybridizations are depicted as red bars. B) Diagnostic PCR for three independent *CCL1* deletion mutants in *F. fujikuroi* (on the left) and *F. graminearum* (on the right). Three independent *CCL1* deletion mutants Δ*ffccl1* in case of *F. fujikuroi* (left panel) and Δ*fgccl1* in case of *F. graminearum* (right panel) show signals for correct homologous integration events of upstream (5‘; *F. fujikuroi*: dia_FfCCL1_5‘//trpC-term, *F. graminearum*: FgCCL1_5’//trpC-term) and downstream (3‘; *F. fujikuroi*: trpC-prom//dia_FfCCL1_3’, *F. graminearum*: trpC-prom//FgCCL1_3’) regions but not for the wild-type gene *CCL1* (*F. fujikuro*i: FfWT_F//FfWT_R, *F. graminearum*: FgWT_F//FgWT_R). M indicates DNA ladder (in this case *Hin*dIII-digested lamdba DNA). C) Southern blot analysis of three independent *CCL1* deletion mutants for *F. fujikuroi* (on the left) and *F. graminearum* (on the right). Genomic DNA was digested with *Sac*I in case of *F. fujikuroi* and *Xba*I in case of *F. graminearum* strains. The downstream (3‘) region and the hygromycin resistance cassette (*hph*) were used for probing in case of *F. fujikuroi* (FfWT: 2.5 kb, Δ*ffccl1*: 7.8 kb) and *F. graminearum* (FgWT: no band, Δ*fgccl1*: 4.3 kb), respectively.

Fig. S3


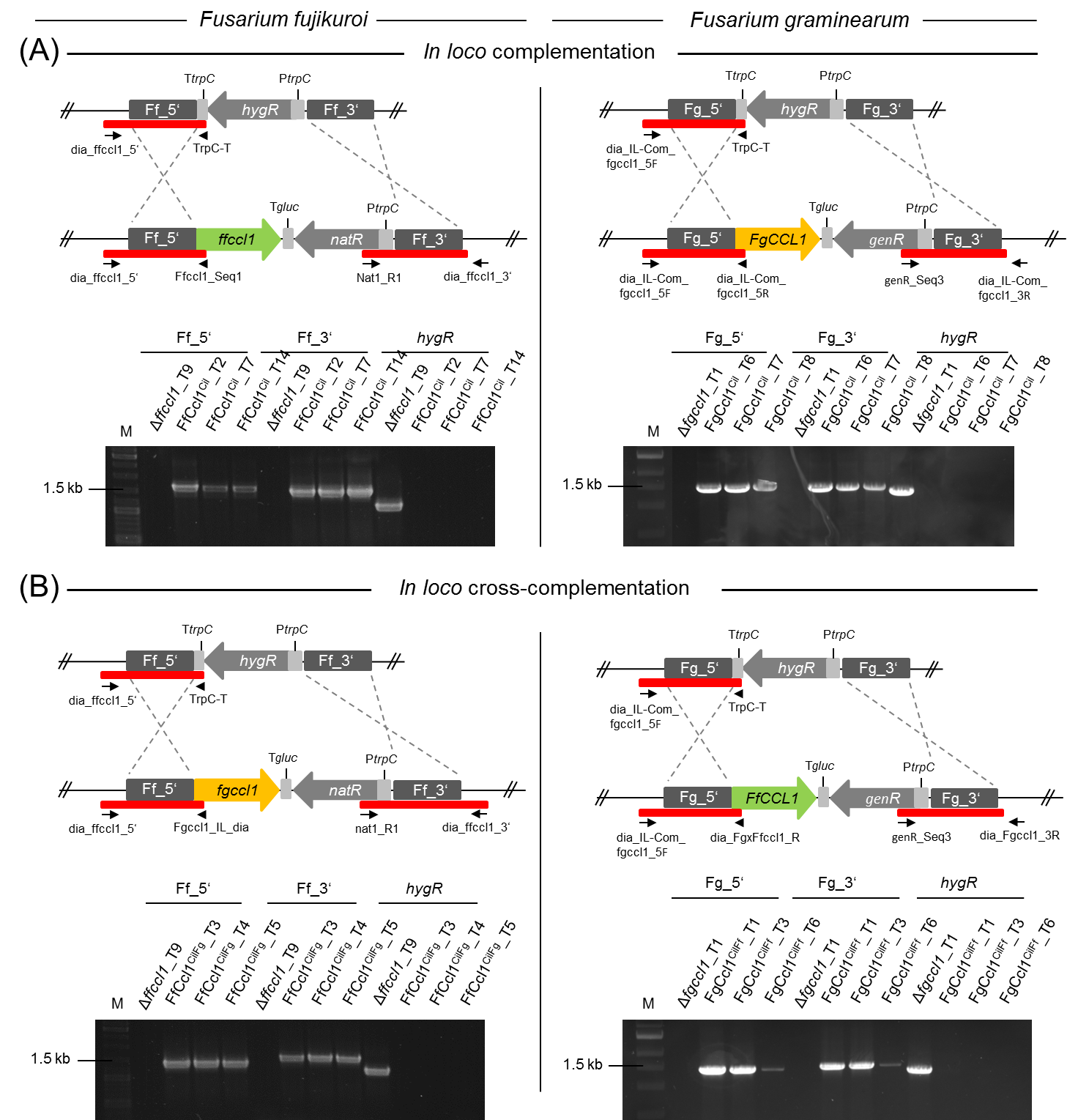


**Fig. S3 Diagnostic PCR verifying complementation (A) and cross-complementation (B) mutants of Δ*ffccl1* and Δ*fgccl1*.** Primers used for diagnostic PCR are highlighted. Expected fragments are depicted as red bars. A) Three independent complementation mutants of Δ*ffccl1* with *FfCCL1* (FfCcl1^Cil^) in case of *F. fujikuroi* (left panel) and of Δ*fgccl1* with *FgCCL1* (FgCcl1^Cil^) in case of *F. graminearum* (right panel) show signals for the correct homologous integration of upstream (5‘; *F. fujikuroi*: dia_ffccl1_5‘//Ffccl1_Seq1, *F. graminearum*: dia_ILCom_FgCCL1_5F//dia_ILCom_FgCCL1_5R) and downstream (3‘; *F. fujikuroi*: dia_ffccl1_3‘//Nat1_R1, *F. graminearum*: gen-Seq3//dia_IL-Com_FgCCL1_3R) regions but not for the hygromycin B resistance cassette (*hygR*, *F. fujikuro*i: dia_ffccl1_5‘//TrpC-T, *F. graminearum*: dia_IL-COM_fgccl1_5F//TrpC-T). B) Three independent cross-complementation mutants of Δ*ffccl1* with *FgCCL1* (Δ*ffccl1/FgCCL1*) in case of *F. fujikuroi* and of Δ*fgccl1* with *FfCCL1* (Δ*fgccl1/FfCCL1*) in case of *F. graminearum* show signals for the correct homologous integration of upstream (5‘; *F. fujikuroi*: dia_ffccl1_5‘// Fgccl1_IL_dia, *F. graminearum*: dia_ILCom_FgCCL1_5F//dia_IL-CCom_FgxFfCCL1_5R) and downstream (3‘; *F. fujikuroi*: dia_ffccl1_3‘//Nat1_R1, *F. graminearum*: gen-Seq3// dia_IL-Com_FgCCL1_3R, 1.55 kb) regions but not for the hygromycin B resistance cassette (*hygR*; *F. fujikuroi*: dia_ffccl1_5‘//TrpC-T, *F. graminearum*: dia_IL_Com_FgCCL1_5F// trpC-T). M: GeneRuler DNA Ladder Mix.

Fig. S4


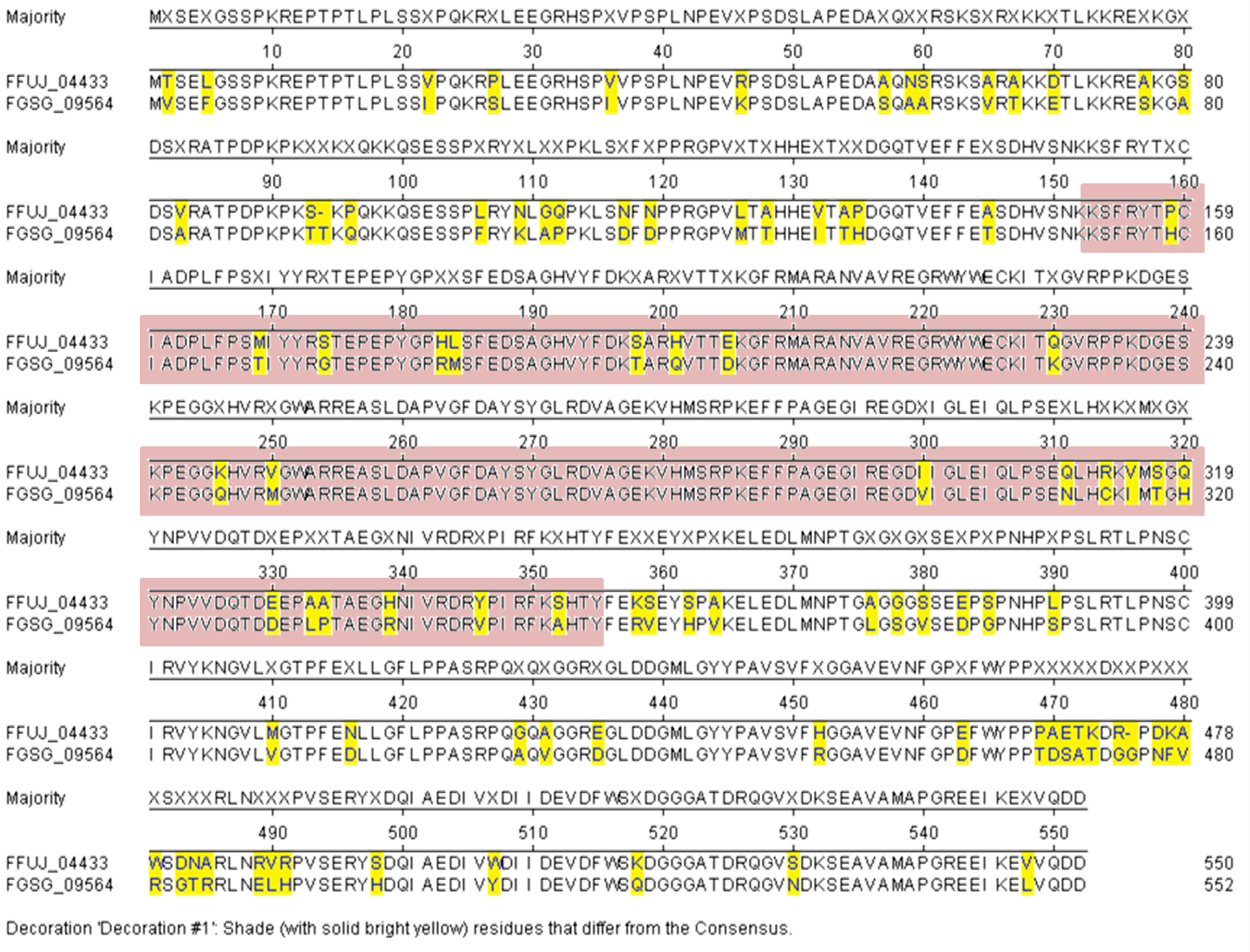


**Fig. S4 Protein alignment of FfCcl1 and FgCcl1.** Protein sequences were analyzed using MegAlign (DNAStar, Madison, Wisconsin, US). The SPRY (SPla and the RYanodine Receptor) domain was identified with the ProSite tool on ExPASy (http://prosite. expasy.org/). Its function is yet unknown, but might involve protein binding (GO:0005515). Residues that differ in both protein sequences are decorated in yellow, SPRY domain is shaded in red.

Fig. S5





**Fig. S5 Infection rates of the Δ*ccl1* mutants compared to the respective *Fusarium* wild-type strains.** For determining the *F. fujikuroi* and *F. graminearum* infection rates germinated rice seedlings (Oryza sativa sp. japonica c.v. Nipponbare) and wheat heads (Triticum aestivum cv. USU-Apogee) were inoculated with the F. fujikuroi wild-type strain and Δffccl1 (left panel) as well as the *F. graminearum* wild type and the Δ*fgccl1* mutant (right panel), respectively, and total gDNA (of the fungus and the plant) was isolated 7 (in case of *F. fujikuroi*) and 21 (in case of *F. graminearum*) days post inoculation. In both cases, five infected plants were combined prior to gDNA extraction. Calculation of the infection rate is based on quantitative PCR of the proportion of fungal gDNA within the fungus/plant gDNA mixture as described in experimental procedures.

Fig. S6


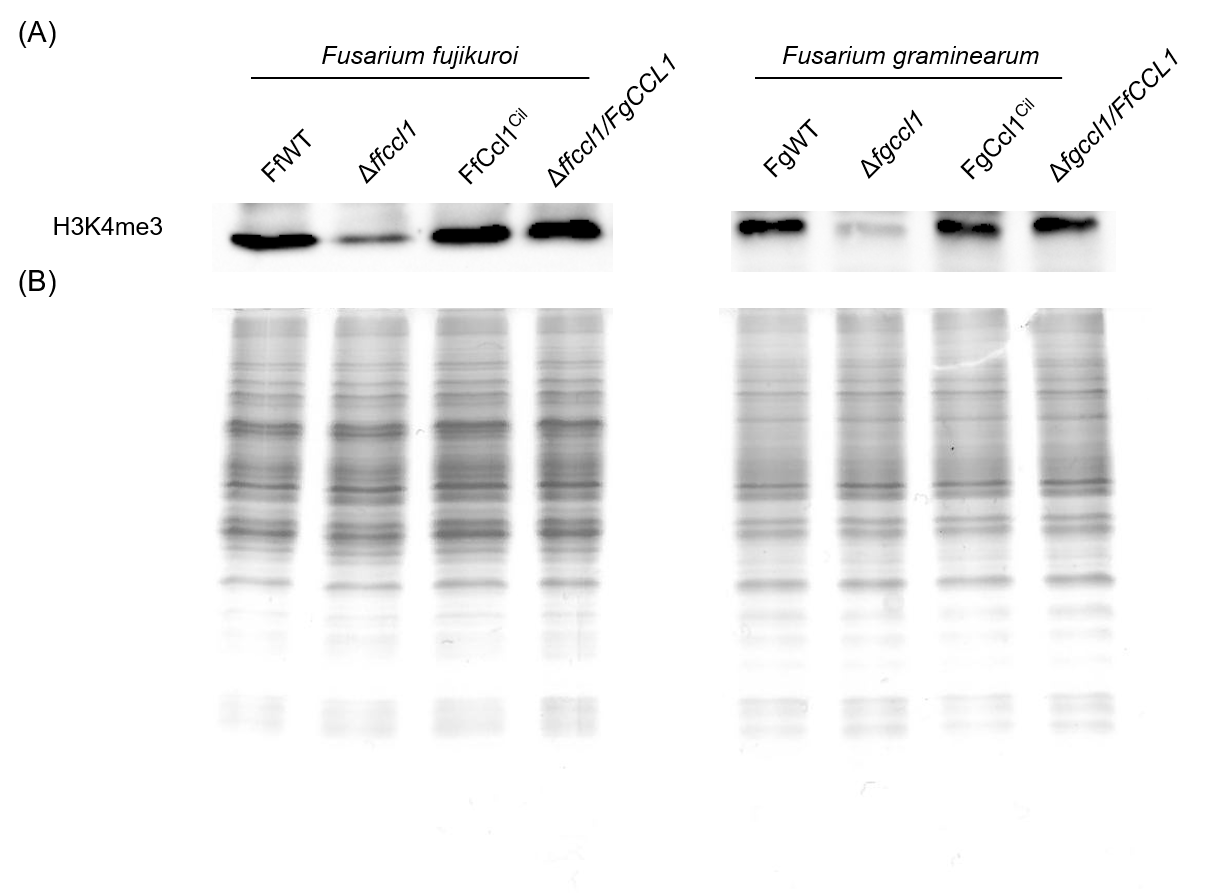


**Fig. S6 Complementation and cross-complementation rescues H3K4me3 levels in Δ*ccl1* strains.** The *Fusarium* wild-type strains (*F. fujikuroi* – FfWT, *F. graminearum* – FgWT), the *CCL1* deletion mutants (*F. fujikuroi* – Δ*ffccl1*, *F. graminearum* – Δ*fgccl1*) as well as the respective complementation (*F. fujikuroi* – FfCcl1^Cil^, *F. graminearum* – FgCcl1^Cil^) and cross-complementation (*F. fujikuroi* – Δ*ffccl1/FgCCL1*, *F. graminearum* – Δ*fgccl1/FfCCL1*) strains were grown for three days on solid complete medium. Whole protein extracts were subsequently isolated from lyophilized mycelia and roughly 15 µg of proteins were used for SDS-Page and western blotting. (A) H3K4me3 antibody ab8580 was used for detection. (B) Coomassie staining was performed as a loading control.

Fig. S7





**Fig. S7 Deletion of *FgCCL1* results in reduced expression of DON genes, while fusarin (FUS) and zearalenone (ZON) genes were upregulated.** The *F. graminearum* wild-type strain (FgWT) was cultivated on potato dextrose agar (PDA) for up to 14 days and mycelium was harvested every 48 h and used for RNA extraction and subsequent cDNA synthesis (left panel). For comparison of FgWT and the Δ*fgccl1* mutant, both strains were grown for 4 days on PDA and mycelia were subsequently harvested for RNA extraction and cDNA synthesis (right panel). To quantify mRNA levels of SM genes, the following primers were used: deoxynivalenol gene cluster (*TRI5*, *TRI6*), fusarin gene cluster (*FUS1*, *FUS9*), zearalenone gene cluster (*PKS13*, *PKS4*) and mRNA levels were related to constitutively expressed reference genes, *i.e.* *FGSG_06257* encoding glyceraldehyde 3-phosphate dehydrogenase, *FGSG_07335* encoding actin and *FGSG_09530* encoding ß-tubulin. Primer efficiencies in the RT-qPCR were kept between 90-110%. Relative expression levels were calculated using the ∆∆Ct method. While fusarin and deoxynivalenol cluster genes were expressed at the highest level 4 days post inoculation (dpi), expression of genes involved in zearalenone biosynthesis were delayed and highest at 8 dpi. Experiments were performed in biological and technical replicates. Mean values and standard deviations are shown. nd, not detectable.

Fig. S8





**Fig. S8 H3K4me3 levels are decreased in the *ccl1* mutants at the highly-decorated actin-encoding genes (*ACN*) in both *Fusarium* spp.** The *F. fujikuroi* wild-type strain (FfWT) and the *CCL1* deletion mutant (Δ*ffccl1*) were grown in synthetic ICI medium with 6 mM glutamine for three days (upper panel). In case of *F. graminearum*, the wild-type (FgWT) and Δ*fgccl1* were grown for 4 days on PDA (lower panel). Mycelium was crosslinked and ChIP assays were conducted using H3K4me2-specific antibodies. Precipitated genomic DNA was quantified by quantitative real-time PCR using primer pairs located in the 5‘ region of *ACN*. Experiments were performed in biological and technical replicates. The amount of precipitated DNA in the respective wild-type strains was arbitrarily set to 1. Mean values and standard deviations are shown. PTMs, post-translational modifications.

Fig. S9





**Fig. S9 Chromatin immunoprecipitation (ChIP) reveals a drastic increase of H3K4 dimethylation at two genes in the gibberellic acid cluster naturally carrying this mark in significant amounts of *F. fujikuroi*.** The *F. fujikuroi* wild-type strain (FfWT) and the *CCL1* deletion mutant (Δ*ffccl1*) were grown in synthetic ICI medium with 6 mM glutamine (gibberellic acid-inducing condition) for three days. Mycelium was crosslinked and used for ChIP analyses. ChIP assays were conducted by using H3K4me2-specific antibodies. Precipitated genomic DNA was quantified by quantitative real-time PCR using primer pairs located in the 5‘ region of *P450-2* and *P450-4*. Experiments were performed in biological and technical replicates. The amount of precipitated DNA in FfWT was arbitrarily set to 1. Mean values and standard deviations are shown.

Fig. S10





**Fig. S10 H3K4me2 levels at SM cluster genes correlated to actin-encoding gene, *ACN*, in *F. fujikuroi.*** The *F.* *fujikuroi* wild-type strain (FfWT) and the *CCL1* deletion mutant (Δ*ffccl1*) were grown in synthetic ICI medium with either 60 mM for fusarin (FUS) or 6 mM glutamine for bikaverin (BIK) and gibberellin (GA) biosynthesis for three days. Mycelium was crosslinked and used for ChIP analyses with H3K4me2-specific antibodies. Precipitated genomic DNA was quantified by RT-qPCR using primer pairs located in the 5‘ region of investigated SM cluster genes. For each SM cluster, two cluster genes were analyzed. Experiments were performed in biological and technical replicates. The amount of precipitated DNA at SM cluster genes was correlated to *ACN*, which was arbitrarily set to 1. Mean values and standard deviations are shown.
